# Vimentin loss inhibits DNA damage responses and promotes cancer cell survival

**DOI:** 10.1038/s44319-026-00913-2

**Published:** 2026-09-04

**Authors:** Elvira Infante, Emmanuel Terriac, Matthieu Gelin, Vanessa Roca, Saline Jabre, Hugo Siegfried, David Pereira, Hugo Varet, Sara Khalilian, Atef Asnacios, Emma J van Bodegraven, Sandrine Etienne-Manneville

**Affiliations:** 1 Cell Polarity, Migration and Cancer Unit, Institut Pasteur, UMR3691 CNRS, Université Paris-Cité, Equipe Labellisée Ligue Contre le Cancer 2023, F-75015 Paris, France; 2 Matières et Systèmes Complexes, Université Paris-Cité, CNRS, F-75013 Paris, France; 3 Bioinformatics and Biostatistics Hub, Institut Pasteur, Université Paris-Cité, F-75015, Paris, France; 4 Department of Translational Neuroscience, UMC Utrecht Brain Center, University Medical Center Utrecht, Utrecht University, 3584 CG Utrecht, The Netherlands

**Keywords:** Cancer, Cell Adhesion, Polarity & Cytoskeleton, DNA Replication, Recombination & Repair

## Abstract

Vimentin intermediate filaments are a hallmark of aggressive tumours and are widely linked to invasion and EMT, yet how vimentin-dependent mechanics shape genome maintenance and therapy response is unclear. Here we show that vimentin, particularly under compressive load, promotes DNA repair competence. In contrast, vimentin-negative cells show impaired DNA damage sensing and downstream signaling, ultimately leading to decreased apoptosis and promoting cell survival under genotoxic stress at the expense of genomic stability. Using controlled cell compression together with genetic and pharmacological perturbations, we find that loss of vimentin in glioblastoma cells limits the expression and activity of core repair pathways because of induced nuclear mechanical compression. Relieving nuclear compression restores DNA damage accumulation and repair kinetics. Functionally, suppression of DNA damage responses enhances survival after clinically relevant DNA-damaging treatments, including temozolomide, X-Ray radiation and cell invasion through tight spaces. These findings invert the prevailing view that vimentin’s contribution to tumour progression stems from enhanced migration and identify a mechanochemical vimentin-nucleus axis that tunes DNA damage responses to favor therapy tolerance and genome evolution.

## Synopsis

We show that vimentin mechanically protects the nucleus from compressive forces, thereby maintaining chromatin organization and DNA damage response (DDR) pathways. Loss of vimentin or increased mechanical compression suppresses DDR signaling, enhances survival after genotoxic stress, and may promote therapy resistance and genomic instability, revealing an unexpected mechanochemical role for vimentin in cancer progression.

- Vimentin preserves DNA damage sensing and repair by shielding the nucleus from microtubule- and LINC-dependent compressive forces.
- Loss of vimentin or mechanical compression remodels chromatin and downregulates major DDR pathways, including ATM, H2AX, Chk1, and 53BP1.
- Impaired DDR in vimentin-deficient cells reduces apoptosis and promotes survival after UV irradiation, X-rays and temozolomide treatment.
- Restoring nuclear shape by disrupting microtubules or the LINC complex rescues chromatin organization and DDR signaling.
- These findings identify a mechanochemical vimentin–nucleus axis that links tumour mechanics to therapy resistance and genomic instability.

## Abstract (web summary)

We show that vimentin mechanically protects the nucleus from compressive forces, thereby maintaining chromatin organization and DNA damage response (DDR) pathways. Loss of vimentin or increased mechanical compression suppresses DDR signaling, enhances survival after genotoxic stress, and may promote therapy resistance and genomic instability, revealing an unexpected mechanochemical role for vimentin in cancer progression.

## Graphical abstract

**Figure:**
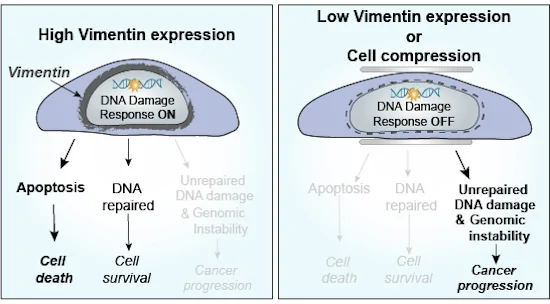

## Introduction

Cancer progression from tumour formation to dissemination involves a complex interplay of genetic, biochemical, and mechanical changes. While genetic and biochemical cues have long been recognised as key drivers, mechanical cues—such as increased tissue stiffness, compressive forces, and shear stresses—have emerged both as significant prognostic factors in cancer progression (Barnes et al, 2018; Martino et al, 2018) and as potential therapeutic targets (Kaukonen et al, 2016; Nia et al, 2020). The contributions of tumour mechanics, encompassing the mechanical properties of both the extracellular environment and tumour cells, are particularly relevant in cancer progression (Linke et al, 2024; Mohammadi and Sahai, 2018).

The mechanics of cells, including cancer cells, rely mainly on their cytoskeleton, composed of actin, microtubules and intermediate filaments. Actin filaments and microtubules are highly conserved cytoskeletal components with a limited number of isoforms and generally maintain a relatively uniform composition across different tissues. In contrast, intermediate filaments (IFs) are encoded by 70 genes—such as vimentin— which can be alternatively spliced (Dutour-Provenzano and Etienne-Manneville, 2021). IF proteins display cell type-specific expression and dynamically adapt to environmental cues, allowing cells to modulate their structural and functional properties in response to physiological and pathological conditions (Etienne Manneville, 2018). Notably, intermediate filament composition can change during processes like cell differentiation or cancer progression. Shifts in protein expression often serve as markers for cellular identity and pathological states and have been linked to patient prognosis (Sharma et al, 2019). The best known example is Vimentin IFs, a major cytoskeletal component of motile mesenchymal cells and a hallmark of epithelial-to-mesenchymal transition (EMT) found in metastatic tumours of epithelial origin (Danielsson et al, 2018; Guo et al, 2012). Beyond serving as biomarkers, IFs play a key role in cellular and tissue mechanics, influencing properties like stiffness, resilience to mechanical stresses, including compressive forces (Etienne-Manneville, 2018; Infante and Etienne-Manneville, 2022; Pogoda et al, 2022; van Bodegraven and Etienne-Manneville, 2021). Changes in IF composition are associated with cell invasive capacity and tumour spreading (Leduc and Etienne-Manneville, 2015), highlighting their key role in the interplay between cell mechanical properties and extracellular mechanical cues. Vimentin expression has been shown to promote cell migration, invasion, and altered mechanics in numerous cancers, including glioblastoma (GBM) (Mendez et al, 2010; Patteson et al, 2019; Sivagurunathan et al, 2022; Strouhalova et al, 2020; Thievessen et al, 2015; van Bodegraven et al, 2025), however, the significance of a potential low- vimentin reservoir within the tumour has never been investigated.

GBMs are the most aggressive and devastating primary brain tumours, with limited treatment options and poor prognoses. As GBM grows in the brain enclosed within a hard skull, compression of the brain tissue and of the tumour mass is a typical feature and a key cause of the clinical symptoms observed in patients with brain cancer. Accumulating evidence from in vitro studies, animal models, and patient tissues demonstrates a clear link between the mechanical properties of tumour tissue and GBM aggressiveness (Barnes et al, 2018; Miroshnikova et al, 2016), as well as increased therapeutic resistance (Erickson et al, 2018). More recently, compression forces were shown to promote GBM progression (Grossen et al, 2022; Khoonkari et al, 2022). The mechanical properties of glioma cells also evolve during tumour progression (preprint: Alibert et al, 2021), suggesting changes in their cytoskeletal composition. It also highlights the need to better understand the dynamic interplay between the mechanics of the cells and their microenvironment, as this relationship has profound implications for tumour progression and therapy resistance.

In this study, we demonstrate the coexistence of both vimentin-positive and vimentin-negative cells within GBM samples and show that while vimentin expression was shown to facilitate GBM cell invasion, its absence enhances cell resilience to genomic stresses. These findings underscore the unexpected dualistic role of vimentin in GBM, suggesting it may serve as a valuable molecular marker for distinguishing tumour cell subpopulations with distinct behaviours. Understanding this heterogeneity is critical for developing more precise and effective therapeutic strategies tailored to the complexity of GBM.

## Results

### Heterogeneous vimentin expression in glioblastoma

**Figure 1:**
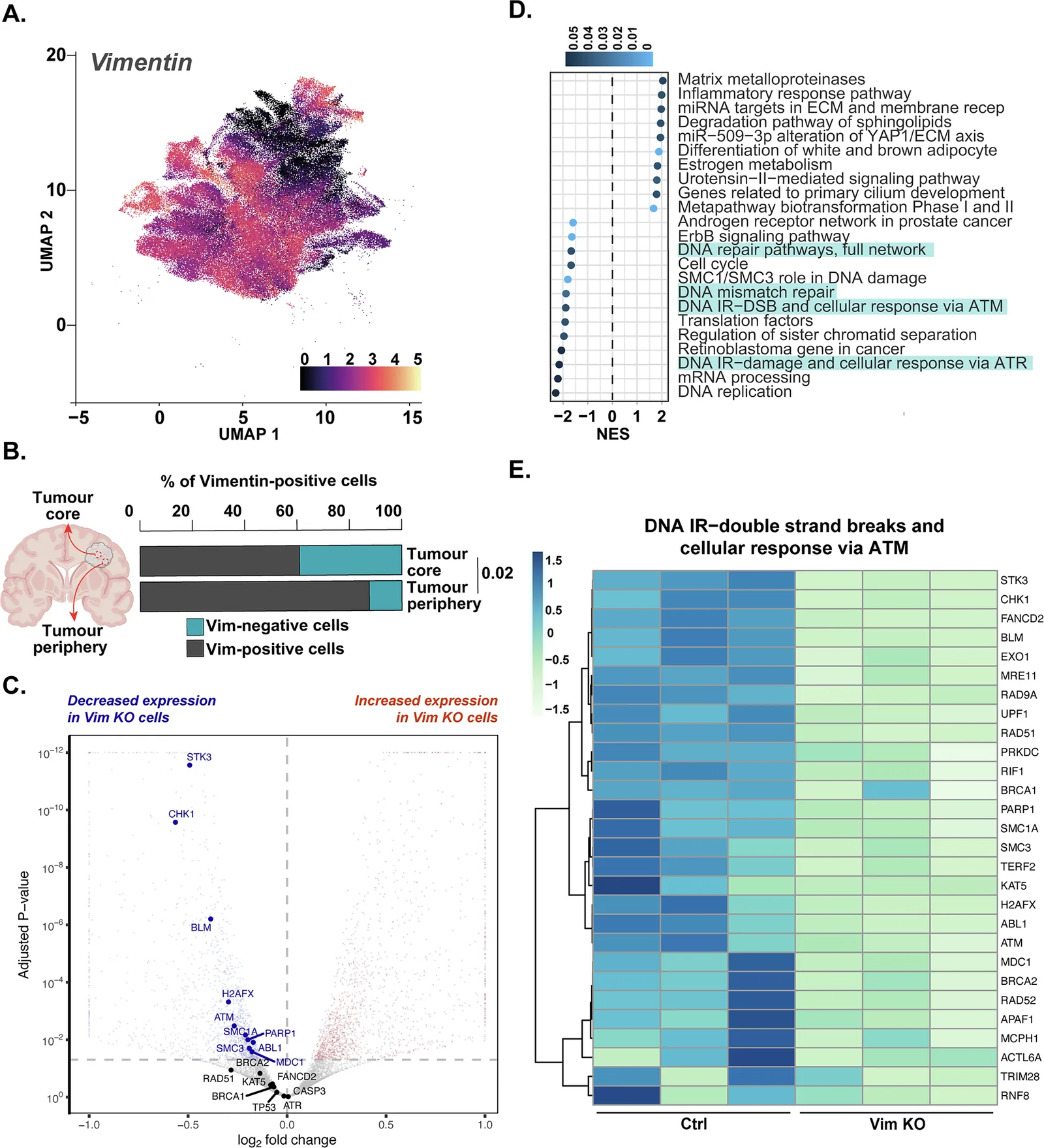
Vimentin heterogeneity in GBM. (**A**) Uniform manifold analysis approximation and projection (UMAP) analysis shows the different neoplastic cell populations present within a large integrated GBM single-cell RNA sequencing dataset. A fire LUT was applied to highlight vimentin heterogeneous expression within these cell populations, with low expression denoted in black and high expression in light yellow. (**B**) Percentage of vimentin-positive neoplastic cells in the tumour core and tumour periphery. Vimentin-positive and -negative neoplastic cell percentages of four (P1–4) different GBM grade IV IDHwt patients for which location information is shown (Darmanis et al, 2017). Data were analysed by the chi-square test of independence. (**C**) Volcano plot of the differential expression analysis between control and vimentin-depleted cells. Differentially expressed (DE) genes were selected based on adjusted *P* value of ≤0.05. The volcano plot is restricted to log2FC between −1 and +1 and adj *P* values >10^(−12)^. Upregulated genes (red dots) and downregulated genes (blue dots) are shown. Data were acquired from three independent experiments; (adjusted) *P* values come from a Wald test on the coefficient of a generalised linear model fitted with the DESeq2 R package. (**D**) Pathway enrichment analysis based on WikiPathways gene-sets using the GSEA method. NES stands for normalised enrichment score. (**E**) Heatmaps providing a visual representation of gene expression of the DNA IR-double-strand breaks and cellular response via ATM pathways in control versus Vim KO U251-MG cells. The heatmap is based on the variance-stabilised transformed count matrix, and rows were re-ordered thanks to a hierarchical clustering using the correlation distance and the Ward aggregation criterion. Gene names are reported. Colour scale ranges from −1.5 to +1.5 as the rows of the matrix have been centred. High expression is denoted in dark blue, while low expression is in light green.

The GBM genetic landscape has been shown to evolve dynamically over space and time, producing an extraordinary degree of cellular complexity and heterogeneity within individual tumours (Greenwald et al, 2024; Qazi et al, 2017; Reinartz et al, 2017; Sottoriva et al, 2013). Analysis of published patient data from single-cell RNA sequencing (scRNAseq) from a large integrated GBM scRNAseq dataset of Isocitrate dehydrogenase (IDH)-wild-type GBM (Ruiz-Moreno et al, 2025a, Data ref: Ruiz-Moreno et al, 2022) showed a high cell-to-cell variation of vimentin expression within the neoplastic cells (Fig. 1A) with significantly more abundant vimentin-negative cells in the core than in the periphery (Fig. 1B) (Darmanis et al, 2017, Data ref: Darmanis et al, 2017). Moreover, the percentage of vimentin-positive cells varies from 0 to 100% across patients (Appendix Fig. S1A). This finding suggests that both vimentin-positive and vimentin–negative cells persist in high-grade tumour and that low or absent vimentin expression may be more advantageous to cells within the tumour core.

### Loss of vimentin impairs DNA damage repair (DDR) pathways in GBM cells

To study whether the absence of vimentin modifies specific cell characteristics, we compared the transcriptome of U251-MG GBM cells expressing high vimentin levels and a vimentin knockout (Vim KO) U251-MG cell population obtained using a CRISPR/Cas9 approach (van Bodegraven et al, 2025) (Appendix Fig. S1B). Transcriptomic analysis revealed a strong modification of gene expression profiles of Vim KO cells compared to control cells (1646 downregulated genes (*p*_adj_ < 0.05) and 1877 upregulated genes (*p*_adj_ < 0.05)) (Fig. 1C). Interestingly, among the most downregulated genes, we found genes belonging to the Gene Ontology (GO) terms related to DNA repair pathways, including DNA repair pathways, full network, DNA mismatch repair, DNA IR (ionising radiation-induced)- DSB (double-strand breaks) and cellular response via ATM (Ataxia telangiectasia mutated) as well as DNA IR−damage and cellular response via ATR (ATM and Rad3 related) (Fig. 1D). A more detailed analysis of the identified genes showed a particularly striking downregulation of genes encoding critical sensors and regulators of the DNA repair machinery in Vim KO cells (Fig. 1E; Appendix Fig. S1C). Notably, this includes genes encoding (1) the H2A histone family member X, H2AX, a key histone variant involved in the detection and signalling of double-strand breaks (DSBs), (2) ATM, a critical kinase which initiates signalling cascades that regulate DSB repair and replication stress response and (3) the checkpoint kinase 1, Chk1, a central kinase in the DNA damage response (DDR) which plays an essential role in cell cycle arrest. Additionally, several *RAD* genes, including *RAD9A*, which can be phosphorylated by ATM in response to DNA damage, and controls the G_1_/S checkpoint(Yin et al, 2004) were also downregulated. A significant downregulation of ABL1, a gene with DNA-binding activity also implicated in DNA damage responses and apoptosis, together with a downregulation of SMC3 and SMC1A involved in DNA repair, was observed in vimentin-deficient cells. The analysis also identified reduced expression of PARP1, a key enzyme in base excision repair (BER) and an important player in the repair of single-strand breaks (SSBs). The downregulation of these genes suggests a substantial impairment of both homologous recombination and non-homologous end joining (NHEJ) pathways in vimentin-deficient cells.

**Figure 2:**
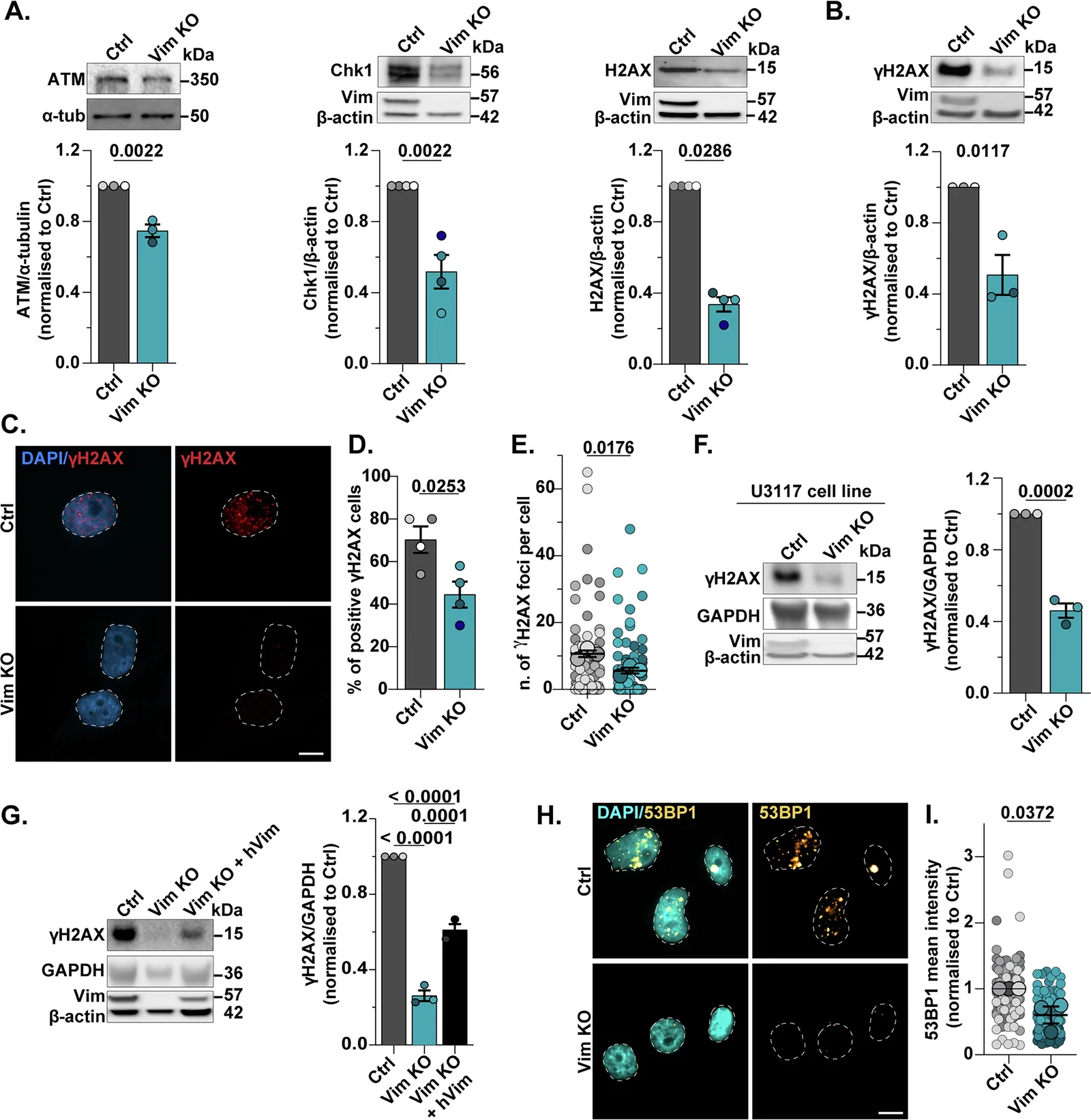
DDR proteins are downregulated in Vim KO cells. (**A**) Expression of ATM, Chk1 and H2AX in control and Vim KO U251-MG cells was analysed by western blot and relative expression was quantified using Fiji. Data were acquired from three independent experiments (ATM), and from four independent experiments (Chk1 and H2AX) and analysed using a two-tailed *t*-test. Error bars represent mean ± SEM. (**B**) Expression of γH2AX in control and Vim KO U251-MG cells was quantified using Fiji. Data were acquired from three independent experiments and were analysed using a two-tailed *t*-test. Error bars represent mean ± SEM. (**C**) U251-MG cells depleted or not for vimentin were plated on glass coverslips and stained for γH2AX and DAPI to visualise the nuclei (scale bar 10 µm). (**D**) The percentage of γH2AX-positive cells was quantified. Data were acquired from four independent experiments and analysed by a two-tailed *t*-test. Error bars represent mean ± SEM. (**E**) SuperPlots of γH2AX foci measured by immunofluorescence. Individual data points are shown as small dots in grey (Ctrl) or blue (Vim KO); the mean of each repeat is shown as larger dots. Data were acquired from three independent experiments and analysed by a two-tailed *t*-test. Error bars represent mean ± SEM. (**F**) Expression of γH2AX in control and Vim KO U3117 cell line was analysed by western blot and quantified using Fiji. Data were acquired from three independent experiments using a two-tailed *t*-test. Error bars represent mean ± SEM. (**G**) Expression of γH2AX in control, Vim KO and Vim KO re-expressing human vimentin (vimentin rescue) were quantified using Fiji. Data were acquired from three independent experiments and analysed by a one-way ANOVA test. Error bars represent mean ± SEM. (**H**) U251-MG cells depleted or not for vimentin were plated on glass coverslips and stained for 53BP1 and DAPI to visualise the nuclei (scale bar 10 µm). (**I**) SuperPlots of the mean intensity of 53BP1 staining. Individual data points are shown as small dots in grey (Ctrl) or blue (Vim KO); the mean of each repeat is shown as larger dots. Data were acquired from three independent experiments and analysed by a two-tailed *t*-test. Error bars represent mean ± SEM.

To confirm the downregulation of major DDR proteins, we performed western blot analysis in Vim KO U251-MG cells. Results showed a significant reduction of ATM, Chk1 and H2AX protein expression compared to control U251-MG cells (Fig. 2A). These observations were also confirmed by transiently downregulating vimentin expression using two different small-interfering RNA (siRNA) technologies (Appendix Fig. S2A). We also validated our observation in patient-derived GBM cells (U3117), which express high levels of vimentin compared to U251-MG and human immortalised astrocytes (Appendix Fig. S2B,C). In these cells, vimentin depletion by CRISPR/Cas9 also reduced the expression level of ATM, Chk1 and H2AX (Appendix Fig. S2C).

DNA double-strand breaks (DSBs) are followed by the phosphorylation of H2AX by kinases such as ATM and ATR (Bonner et al, 2008). This newly phosphorylated protein, γH2AX, is the first step in recruiting DNA repair proteins. Western blots as well as immunofluorescence experiments showed a decrease in γH2AX in Vim KO U251-MG cells (Appendix Fig. S2B–E), in patient-derived GBM cells (U3117) (Fig. 2F), and in U251-MG siRNA-mediated vimentin-depleted cells compared to control cells (Appendix Fig. S2D). This result was also confirmed in five different single clones of Vim KO U251-MG obtained using CRISPR/Cas9 (Appendix Fig. S2E). Moreover, re-expression of human vimentin in Vim KO U251-MG cells led to a partial but highly significant rescue of γH2AX (Fig. 2G).

Following activation of the DNA damage response (DDR) by ATM, Nuclear factor kappaB (NF-κB) is recruited to the nucleus, leading to the transcription of genes involved in cell survival, inflammation, and repair (Miyamoto, 2011). We observed a decrease in nuclear NF-κB in vim KO cells compared to control cells (Appendix Fig. S2F). Finally, we also checked the expression level of the p53-binding protein 1, 53BP1, a crucial component of DNA DSB signalling and repair, which surprisingly was not included in the GO list of DNA repair pathway genes. Immunofluorescence analysis showed that 53BP1 was also strongly reduced in Vim KO cells, further confirming the general inhibition of DNA damage sensing and DDR pathways (Fig. 2H,I). Together, these results show a profound alteration of the DDR pathway in cells lacking vimentin and suggest that vimentin-negative GBM cells have hindered capacity to detect and efficiently repair DNA damage.

### Loss of vimentin impairs DNA damage repair (DDR) pathways in adenocarcinoma cells

To determine if vimentin expression also influenced DNA repair gene expression in the context of epithelial-to-mesenchymal transition, we used a breast adenocarcinoma cell line (MDA-MB-231). These cells lack epithelial markers like E-cadherin and instead express mesenchymal markers such as vimentin. Knockdown of vimentin using siRNA led to the downregulation of DNA damage response (DDR) proteins, including Chk1 and H2AX (Appendix Fig. S2G), consistent with observations in GBM cells. Finally, downregulation of γH2AX was also observed in vimentin-depleted MDA-MB-231 cells (Appendix Fig. S2H).

This demonstrates that vimentin’s impact on DDR pathway regulation is observed across different cellular contexts, including tumours of epithelial origin.

### Loss of vimentin impairs cell responses to DNA damage

**Figure 3:**
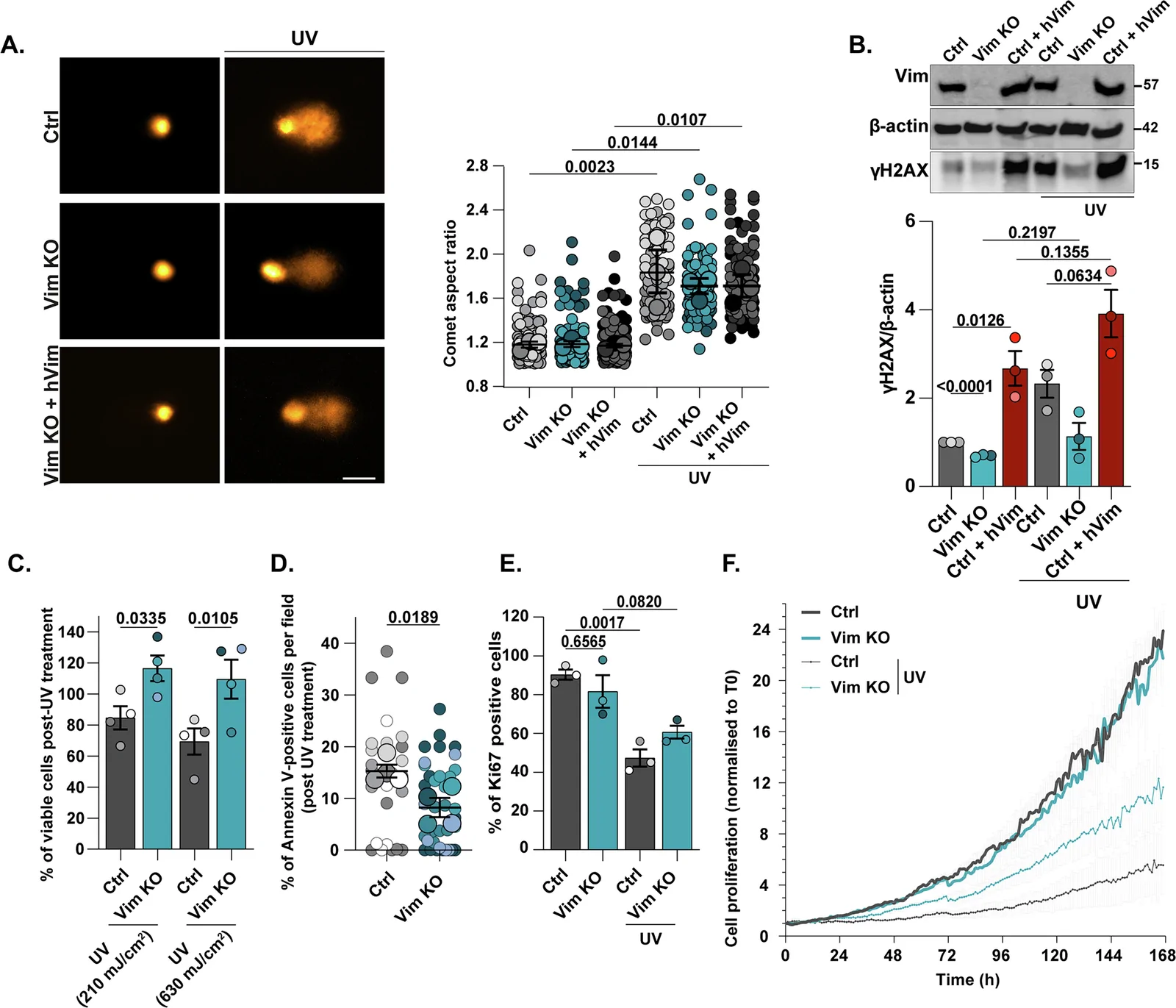
Vimentin-depleted cells are less sensitive to DNA damage. (**A**) (Left) Representative images of U251-MG control, vimentin-depleted cells and vimentin-depleted cells overexpressing human vimentin were irradiated or not with 1.05 J/cm^2^ UV light and after 3 h subjected to alkaline comet assay (scale bar 20 µm). (Right) SuperPlots of the comet aspect ratio. Individual data points are represented as small dots in grey (Ctrl), blue (Vim KO) or black (Vimentin rescue). The mean of each repeat is shown as larger dots. Data were acquired from three independent experiments and analysed by a one-way ANOVA test. Error bars represent mean ± SEM. (**B**) Control, Vim KO and control U251-MG cells overexpressing human vimentin were irradiated or not with 210 J/cm^2^ UV light. Expression of γH2AX was analysed by western blot and quantified using Fiji. Data were acquired from three independent experiments using a two-tailed *t*-test. Error bars represent mean ± SEM. (**C**) MTT assay of control and Vim KO cells was performed 24 h after 210 or 630 mJ/cm^2^ UV light irradiation. Data were acquired from four independent experiments and analysed by a one-way ANOVA test. Error bars represent mean ± SEM. (**D**) Quantification of the percentage of annexin V-positive cells in control and Vim KO U251 cells after 24 h of UV light irradiation. SuperPlots of the percentage of Annexin V-positive cells. Individual data points are represented as small dots in grey (Ctrl) or blue (Vim KO). The mean of each repeat is shown as larger dots. Data were acquired from three independent experiments and analysed by a two-tailed *t*-test. Error bars represent mean ± SEM. (**E**) Quantification of ki67-positive cells 24 h after 210 mJ/cm^2^ UV light irradiation. Data were acquired from three independent experiments and analysed by a one-way ANOVA test. Error bars represent mean ± SEM. (**F**) The proliferation kinetics of control and Vim KO U251-MG cells after 210 mJ/cm^2^ UV light irradiation was monitored for 7 days by Holomonitor cell proliferation assay. Five positions of two separate wells were acquired per condition. Data were from three independent experiments and analysed by a one-way ANOVA test. Error bars represent mean ± SEM.

To determine if the DNA damage-sensing machinery was functionally altered in vimentin-deficient cells, we artificially induced DNA damage by exposing control and Vim KO U251-MG cells to ultraviolet (UV) radiation, a well-established and potent inducer of DNA lesions. To directly assess DNA damage, we performed a comet assay under alkaline conditions, which detects both single-strand and double-strand breaks (Olive and Banath, 2006). UV treatment significantly increased the comet aspect ratio in control, Vim KO and vimentin-rescued cells with no significant differences between conditions (Fig. 3A), indicative of DNA break formation regardless of the presence or absence of vimentin. However, while in control U251-MG cells, γH2AX levels increased markedly following UV exposure, as expected, reflecting the activation of the DNA damage response, Vim KO cells exhibited a significantly attenuated increase in γH2AX, confirming impaired DNA damage sensing (Fig. 3B). In contrast, γH2AX levels were dramatically increased when vimentin was overexpressed in U251-MG cells both in control conditions and after UV exposure (Fig. 3B). Given this strong influence of vimentin expression on DNA damage sensing and DDR pathways, we next examined its impact on GBM cell survival following DNA damage. Western blot analysis of the proliferation marker PCNA revealed comparable baseline expression between non-treated control and Vim KO cells. In control cells, PCNA levels significantly dropped following UV exposure, whereas in Vim KO cells, UV exposure did not induce any statistically significant changes in PCNA levels (Appendix Fig. S2I).

Quantification of cell viability using an MTT assay revealed significantly higher survival rates of Vim KO cells compared to control cells following UV exposure (Fig. 3C). Additionally, the percentage of cells positive for the apoptosis marker Annexin V was significantly lower in Vim KO cells (Fig. 3D), consistent with reduced apoptosis and enhanced cell viability. Ki67 staining and monitoring of cell population growth over seven days post-UV exposure further confirmed this trend: while UV-induced DNA damage effectively halted the expansion of control cells, it only partially reduced the proliferation of Vim KO cells (Fig. 3E,F).

Together, these findings demonstrate that vimentin is important for the induction of DDR pathways, including DNA damage-induced inhibition of cell proliferation and induction of apoptosis. Loss of vimentin allows GBM cells to bypass DNA damage checkpoints, thus contributing to enhanced cell survival and proliferation under conditions of genotoxic stress.

### Vimentin IFs protect the GBM cell nucleus from microtubule- and LINC complex-dependent compression

**Figure 4:**
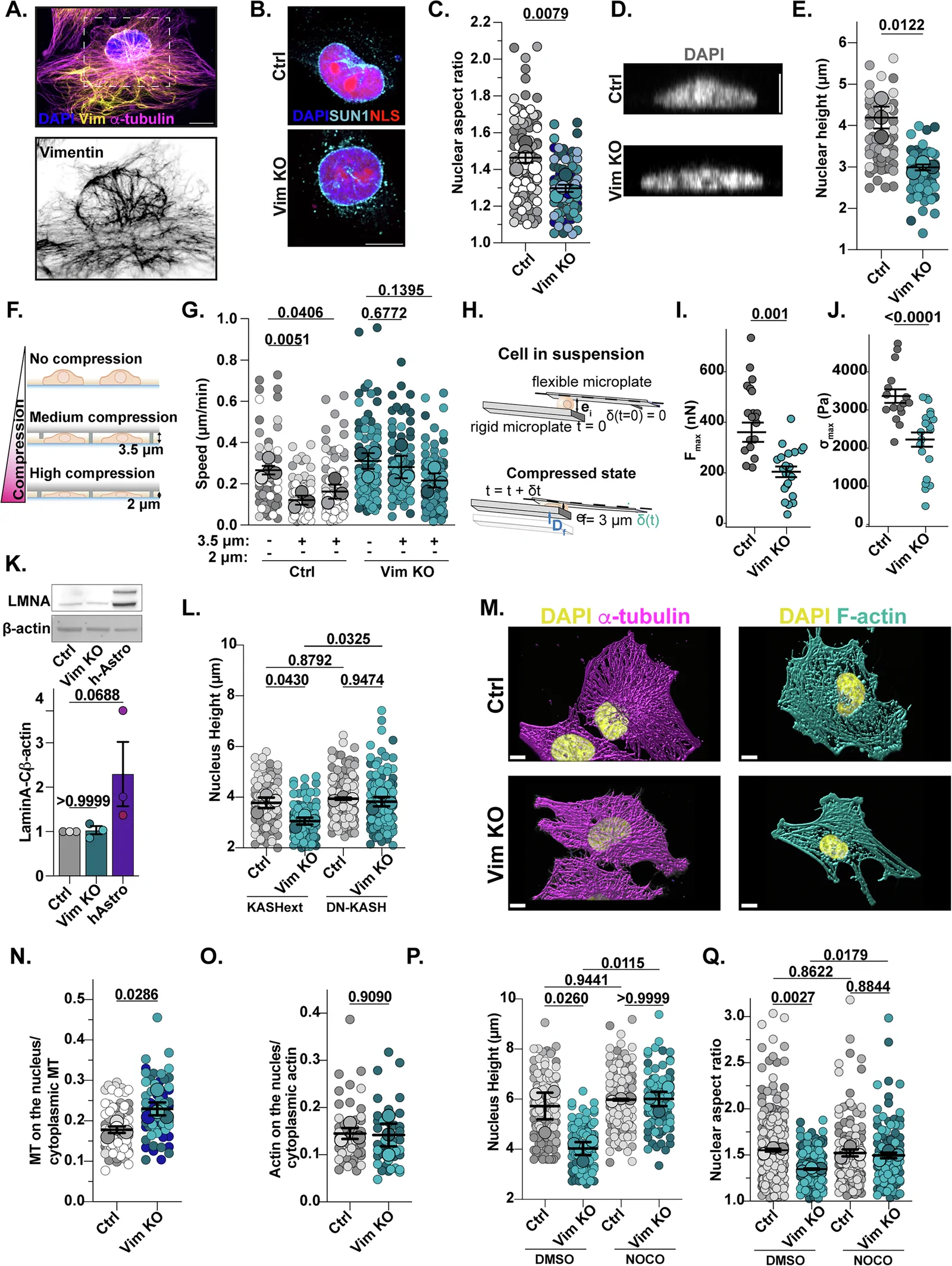
Vimentin perinuclear cage mechanically protects against microtubule and LINC complex-dependent nuclear compression. (**A**) (Left) Representative maximum intensity projection of confocal z-stacks of U251-MG cells plated on glass coverslips and stained for vimentin, a-tubulin and DAPI. (Right) Inset of inverted LUT of boxed region showing vimentin staining around the nucleus (scale bar 10 µm). (**B**) Representative maximum intensity projection of confocal images of NLS-mCherry control or Vim KO U251-MG cells plated on glass coverslips and stained for SUN1 and DAPI (scale bar 10 µm). (**C**) SuperPlots of nuclear aspect ratio. Individual data points are represented as small dots in grey (Ctrl) or blue (Vim KO). The mean of each repeat is shown by bigger dots. Data were acquired from four independent experiments and analysed using a two-tailed *t*-test. Error bars represent mean ± SEM. (**D**) Representative orthogonal view or z-stacks acquired by confocal microscopy of control and Vim KO U251-MG cells. (**E**) SuperPlots of nuclear aspect ratio. Individual data points are represented as small dots in grey (Ctrl) or blue (Vim KO). The mean of each repeat is shown by bigger dots. Data were acquired from three independent experiments and analysed using a two-tailed *t*-test. Error bars represent mean ± SEM. (**F**) Representative scheme of the cell confiner device. (**G**) SuperPlots of migration speed of control or Vim KO under no, medium (~3.5 µm) or high (~2 µm) compression (Movies EV1 and EV2). Individual data points are represented as small dots. The mean of each repeat is shown by bigger dots. Data were acquired from three independent experiments and analysed using a one-way ANOVA test. Error bars represent mean ± SEM. (**H**) Schematic of the microplate rheology used to deform a cell in suspension from its original size to 3 µm thickness and measure the (**I**) maximal force and (**J**) stress needed for this deformation. Data were acquired from three independent experiments and analysed using a two-tailed *t*-test. Error bars represent mean ± SEM. (**K**) Expression of lamin A/C in control, Vim KO U251-MG cell line and in human astrocytes was analysed by western blot and quantified using Fiji. Data were acquired from three independent experiments and analysed using the Kruskal–Wallis test. Error bars represent mean ± SEM. (**L**) SuperPlots of nuclear height in Ctrl or Vim KO U251-MG cells stably expressing KASHext (left) or DN-KASH (right). Individual data points are shown as small dots in grey (Ctrl) or blue (Vim KO); the mean of each repeat is indicated by larger dots. Data were acquired from three independent experiments and analysed by one-way ANOVA. Error bars represent mean ± SEM. (**M**) Representative 3D reconstructions generated using IMARIS software from confocal z-stacks of control and Vim KO U251-MG cells plated on glass coverslips and stained for α-tubulin and DAPI (Left) or F-actin and DAPI (Right) (scale bar: 8 µm). (**N**) SuperPlots of the ratio of microtubule intensity between the nucleus and the cytoplasm. Individual data points are represented as small dots in grey (Ctrl) or blue (Vim KO). The mean of each repeat is shown by bigger dots. Data were acquired from four independent experiments and analysed using a two-tailed *t*-test. Error bars represent mean ± SEM. (**O**) SuperPlots of the ratio of actin intensity between the nucleus and the cytoplasm. Individual data points are represented as small dots in grey (Ctrl) or blue (Vim KO). The mean of each repeat is shown by bigger dots. Data were acquired from four independent experiments and analysed using a two-tailed *t*-test. Error bars represent mean ± SEM. (**P**) SuperPlots of nuclear height in Ctrl or Vim KO U251-MG cells treated for 24 h with DMSO (left) or Nocodazole (NOCO, right). Individual data points are shown as small dots in grey (Ctrl) or blue (Vim KO); the mean of each repeat is indicated by larger dots. Data were acquired from three independent experiments and analysed by one-way ANOVA. Error bars represent mean ± SEM. (**Q**) SuperPlots of nuclear aspect ratio in Ctrl or Vim KO U251-MG cells treated for 24 h with DMSO (left) or Nocodazole (right). Individual data points are shown as small dots in grey (Ctrl) or blue (Vim KO); the mean of each repeat is indicated by larger dots. Data were acquired from three independent experiments and analysed by one-way ANOVA. Error bars represent mean ± SEM.

The well-established mechanical role of vimentin IFs (Infante and Etienne-Manneville, 2022) (van Bodegraven et al, 2025) and the significant impact of vimentin depletion on gene expression detailed in this study (Fig. 1C) led us to hypothesise that cytoplasmic vimentin IFs might directly influence nuclear shape and organization. In U251-MG GBM cells, vimentin filaments form a distinct perinuclear cage structure (Fig. 4A), consistent with observations in other cell types (Patteson et al, 2019). Despite no significant changes in the nuclear volume between control and Vim KO cells (Appendix Fig. S4A), the nuclear morphology was strongly affected. In Vim KO cells plated on glass coverslips, nuclei exhibited increased circularity (as indicated by a decreased aspect ratio) and were significantly flatter (reduced height) compared to control cells (Fig. 4B–E). Re-expression of vimentin in Vim KO cells rescued the nuclear height (Appendix Fig. S3B), confirming the role of vimentin in the control of nuclear shape.

To confirm nuclear flattening of vim KO cells in live cells, we assessed their migratory behaviour under vertical confinement. Cells were placed in a static cell confiner (Le Berre et al, 2014) made of two glass coverslips separated by 3.5 or 2 µm, generating “medium” or “high” compression, respectively (Fig. 4F). In contrast to other cell types (Lomakin et al, 2020), fibronectin coating of the lower coverslip was required for GBM cell survival and migration in these compressive conditions. Both medium and high compression substantially reduced the migration speed of control cells, which have a nuclear height of more than 4 μm (Fig. 4G; Movie EV1). In contrast, these compression conditions did not significantly affect the migration speed of in Vim KO cells, which have a nuclear height of less than 3 μm (Fig. 4G; Movie EV2). Interestingly, Vim KO cells migrated faster than control cells in a vertically confined environment, suggesting that vimentin depletion induces nuclear and cell flattening.

Finally, we confirmed that the impact of vimentin loss on nuclear morphology was associated with a change in cell mechanical properties. We used single-cell microplate rheology, trapping individual cells between two microplates (Alibert et al, 2021), to mechanically compress the cells from their original suspension state (~10–15-µm thickness) to a final state of 3-µm thickness and measured the force required to generate such deformation (Fig. 4H). Both the maximum force and stress-strain values required to achieve this deformation were markedly lower in Vim KO cells compared to control cells (Fig. 4I,J), confirming that Vim KO cells are more easily compressed than control cells, probably because of their flatter nucleus.

Although our previous observation showed that the intrinsic mechanical properties of the nucleus not affected by vimentin loss (van Bodegraven et al, 2025), we checked if vimentin depletion may affect lamin A/C expression. Lamin A expression, which is particularly low in glial cells (Jung et al, 2012), appeared even lower in GBM cells compared to human astrocytes and was not significantly modified in Vim KO cells (Fig. 4K), strongly suggesting that nuclear shape changes observed in Vim KO cells are not due to an alteration of lamins. Altogether, our findings suggest that the cytoplasmic vimentin network controls nuclear shape not by affecting nuclear mechanics per se but by shielding the nucleus from perinuclear compressive forces.

Since IFs interact with the nuclear envelope through the linker of nucleoskeleton and cytoskeleton (LINC) complex, we investigated if perturbation of the LINC complex could mimic the depletion of vimentin intermediate filaments. We generated WT and Vim KO cell lines stably expressing a dominant negative KASH domain (DN-KASH), which is known to interfere with the nesprin-Sun interaction in parallel to a similar construct (KASHext) with a C-terminal extension that prevents the binding to SUN (Appendix Fig. S3C) (Infante et al, 2018; Stewart-Hutchinson et al, 2008). DN-KASH expression had no effect on the nuclear aspect ratio and height in control cells. However, it rescued the nuclear aspect ratio and height of Vim KO cells to similar values as control cells (Fig. 4L; Appendix Fig. S3D). These results indicate that the LINC complex is not required for vimentin-mediated mechanical protection of the nucleus, but that it contributes to the transmission of mechanical compressive forces onto the nucleus when vimentin filaments are absent. Thus, we assessed whether the lack of vimentin IF modified other cytoskeletal network in proximity to the nucleus. Analysis of confocal images showed a clear increase in microtubules on the nucleus, but no clear changes in the level of F-actin (Fig. 4M–O). To determine whether this accumulation of microtubules on the nucleus is responsible for the decreased nuclear height of Vim KO cells, we treated cells with nocodazole (NOCO). Microtubule-depolymerisation with nocodazole rescued the nuclear height and aspect ratio of Vim KO cells (Fig. 4P,Q). By contrast, treatment with a ROCK inhibitor (GSK269962A, 5 μM) or blebbistatin (200 nM or 2 μM) to disrupt actomyosin contractility did not restore nuclear shape (Appendix Fig. S3E–G), indicating that actomyosin-generated forces are not responsible for the vimentin-depletion–induced changes in nuclear shape. Consistently, partial disruption of actin polymerisation with low doses of cytochalasin D (100 or 200 nM; to prevent cell detachment) also failed to rescue nuclear aspect ratio or height in Vim KO cells. These results strongly suggest that the changes in nuclear shape observed in Vim KO cells are mainly due to an accumulation of microtubules over the nucleus and their interactions with the LINC complex.

Taken together, these results suggest that the vimentin network shields the nucleus from microtubule-dependent compressive forces transmitted through the LINC complex to the nuclear envelope. We hypothesised that vimentin IFs may thus influence nuclear mechanotransduction in response to compressive forces.

### Cell compression mimics the effects of vimentin loss on DDR pathways

**Figure 5:**
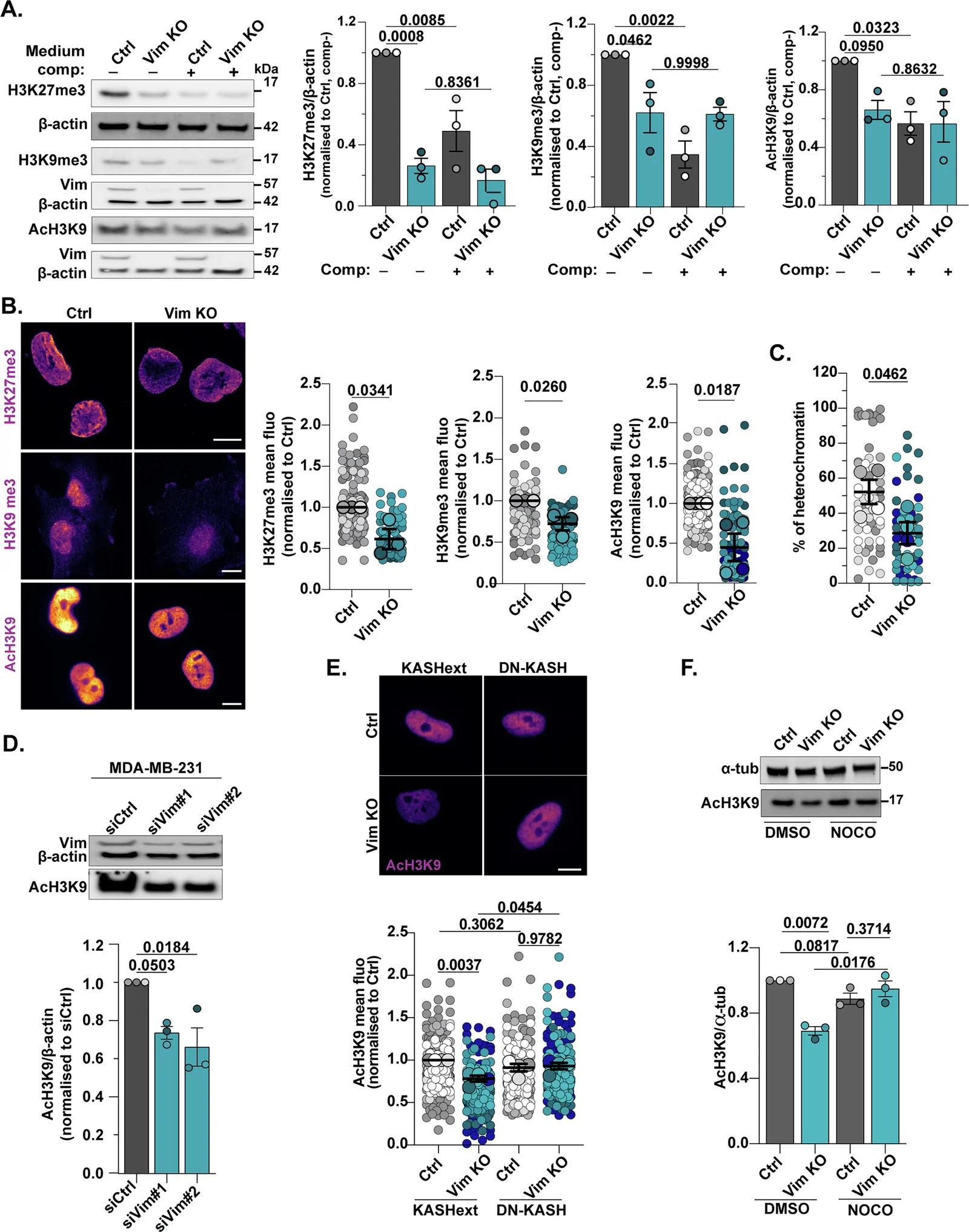
Cell compression mimics the effect of vimentin depletion on chromatin modifications. (**A**) Expression of H3K27me3, H3K9me3 and AcH3K9 in control and Vim KO U251-MG cells subjected to 4 h of medium compression or no compression was analysed by western blot. Relative protein expression levels were quantified using Fiji. Data were acquired from three independent experiments and analysed using a one-way ANOVA. Error bars represent mean ± SEM. (**B**) (Top panels). Representative maximum intensity projection of confocal z-stacks of control or Vim KO U251-MG cells plated on glass coverslips and stained for H3K27me3 and representative epifluorescence images (medium and bottom panels) of H3K9me3 and AcH3K9 in control and Vim KO U251-MG, with relative mean intensity quantification. Data were represented as SuperPlots. Individual data points are represented as small dots in grey (Ctrl) or blue (Vim KO). The mean of each repeat is symbolised by bigger dots. Data were acquired from three independent experiments and four independent experiments for AcH3K9 and analysed using a two-tailed *t*-test. Error bars represent mean ± SEM. (**C**) SuperPlots of the percentage of heterochromatin per nucleus. Individual data points are represented as small dots in grey (Ctrl) or blue (Vim KO). The mean of each repeat is shown by bigger dots. Data were acquired from four independent experiments and analysed using a two-tailed *t*-test. Error bars represent mean ± SEM. (**D**) Expression of vimentin and AcH3K9 in MDA-MB-231 cells depleted or not for vimentin using siRNA technology was analysed by western blot analysis, and relative protein expression quantified using Fiji. Data were acquired from three independent experiments and analysed using a two-tailed *t*-test. Error bars represent mean ± SEM. (**E**) (Top). Representative maximum intensity projection of confocal z-stacks of Ctrl or Vim KO U251-MG cells stably expressing KASHext or DN-KASH and stained for AcH3K9. (Bottom). SuperPlots of AcH3K9 fluorescence intensity in Ctrl or Vim KO U251-MG cells stably expressing KASHext (left) or DN-KASH (right). Individual data points are shown as small dots in grey (Ctrl) or blue (Vim KO); the mean of each repeat is indicated by larger dots. Data were acquired from three independent experiments and analysed by one-way ANOVA. Error bars represent mean ± SEM. (**F**) Expression of AcH3K9 in control and Vim KO treated for 4 h with DMSO (vehicle) or Nocodazole and quantified using Fiji. Data were acquired from three independent experiments and analysed by a one-way ANOVA test. Error bars represent mean ± SEM.

Mechanical stress is known to alter chromatin organization and induce DNA damage (Jain et al, 2013; Le et al, 2016; Nava et al, 2020; Shah et al, 2021). We thus investigated whether vimentin depletion, which results in nuclear flattening, had an effect on histone marks and chromatin organization. Post-translational modifications of histone 3 (H3), including lysine 27 and lysine 9 tri-methylation (H3K27me3 and H3K9me3) and lysine 9 acetylation (AcH3K9), were markedly reduced in Vim KO cells compared to control cells (Fig. 5A), without any alteration of H3 expression level (Appendix Fig. S4A). Immunofluorescence analysis of Vim KO cells confirmed the results of the western blot experiments (Fig. 5B). Analysis of chromatin staining showed a marked reduction of heterochromatin in Vim KO cells (Fig. 5C), which may explain the overall increase in gene expression in Vim KO compared to WT cells (Fig. 1C (1648 genes significantly downregulated and 1877 significantly upregulated). Re-expressing human vimentin in Vim KO cells, which rescues nuclear height (Appendix Fig. S3B) rescued H3K27me3 expression to a similar extent as control condition (Appendix Fig. S4B). Vimentin siRNA also led to an inhibition of histone marks (Appendix Fig. 4C).

Moreover, the downregulation of AcH3K9 was also observed in vimentin-depleted MDA-MB-231 cells, indicating that vimentin loss exerts a comparable effect on histone marks across different cellular contexts (Fig. 5D).

We then asked whether nuclear mechanical compression could mimic the effects observed following vimentin depletion. Cell compression with the cell confiner system (3.5 µm height) reduced H3 post-translational modifications in control cells, mimicking the effect of vimentin depletion (Fig. 5A). In contrast, this compression had no significant effect on Vim KO cells, whose nucleus is generally flatter than 3.5 µm (Fig. 5A). Expression of the dominant negative DN-KASH construct or treatment with the microtubule depolymerising drug nocodazole, both of which rescued nuclear height, also rescued AcH3K9 levels (Fig. 5E, F). By contrast, ROCK inhibition, which did not rescue nuclear shape, also did not rescue H3K27 methylation in Vim KO cells (Appendix Fig. S4D). We also verified that lamin A depletion using siRNA did not impact histone modification, confirming that the effects of vimentin loss are not due to a change in cell contractility or a decrease in lamin A expression (Appendix Fig. S4E).

These results suggest that in the absence of vimentin IFs, microtubule and LINC complex-dependent increased compression of the nucleus causes significant chromatin modifications, likely responsible for the changes in gene expression observed in Vim KO cells.

**Figure 6:**
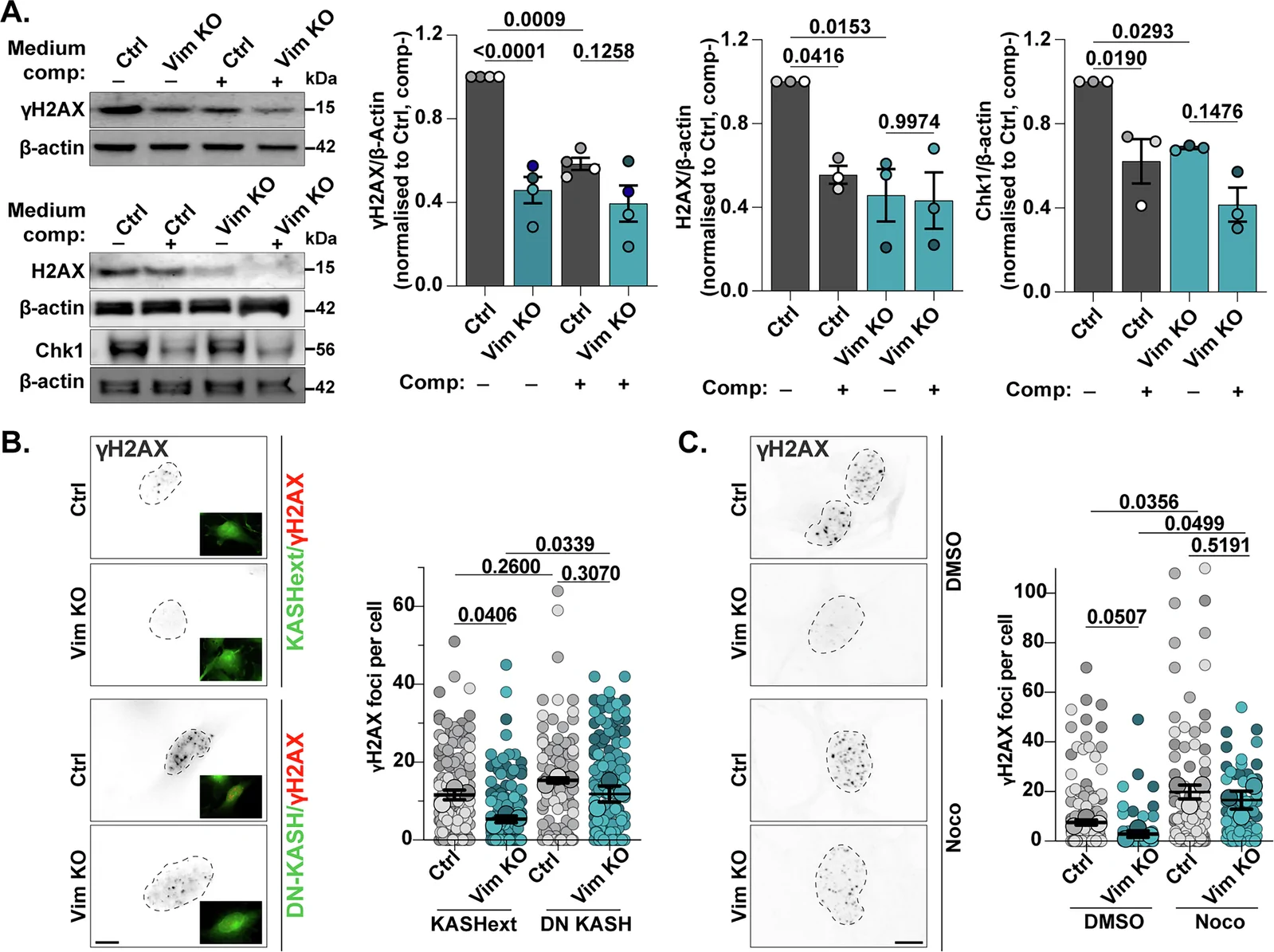
Cell and nuclear compression mimics the effect of vimentin depletion on DNA damage signalling. (**A**) Expression of γH2AX, H2AX and Chk1 proteins in control and Vim KO U251-MG cells undergoing 16 h of medium compression or no compression was analysed by Western blot, and relative expression quantified using Fiji. Data were acquired from four independent experiments and analysed using a one-way ANOVA test. Error bars represent mean ± SEM. (**B**) Representative images of Ctrl or Vim KO U251-MG cells stably expressing KASHext or DN-KASH (insets) and stained for γH2AX. SuperPlots show γH2AX foci in Ctrl or Vim KO U251-MG cells stably expressing KASHext (left) or DN-KASH (right). Individual data points are shown as small dots in grey (Ctrl) or blue (Vim KO), and the mean of each independent experiment is indicated by larger dots. Data were acquired from three independent experiments and analysed using a one-way ANOVA. Error bars represent mean ± SEM. (**C**) Representative images of U251-MG Ctrl or Vim KO cells treated with either DMSO (vehicle) or Nocodazole for 24 h and stained for γH2AX and DAPI to visualise the nuclei (scale bar 10 µm). SuperPlots show γH2AX foci in Ctrl or Vim KO U251-MG treated with DMSO (vehicle) or Nocodazole (right). Individual data points are shown as small dots in grey (Ctrl) or blue (Vim KO), and the mean of each independent experiment is indicated by larger dots. Data were acquired from three independent experiments and analysed using a two-tailed *t*-test. Error bars represent mean ± SEM.

We next evaluated whether mechanical compression of the nucleus could also mimic the effects of vimentin depletion on DDR pathways. Compression of control cells using the static cell confiner led to downregulation of γH2AX, H2AX, and Chk1 protein levels, mirroring the effects observed in Vim KO cells (Fig. 6A). Expression of the dominant negative DN-KASH construct or treatment with the microtubule depolymerising drug nocodazole (NOCO), which both rescued nuclear height and AcH3K9 levels, also rescued the γH2AX expression (Fig. 6B,C). These results suggest that in the absence of vimentin IFs, microtubule and LINC complex-dependent increased compression of the nucleus plays an important role in the decreased expression of DDR-related genes observed in Vim KO cells.

To further evaluate the impact of compressive stress, we compared DDR protein expression in U251-MG cells cultured as adherent monolayers (2D) versus in suspension. In suspension, γH2AX levels were strongly increased in Vim KO cells relative to adherent culture (Appendix Fig. S5A), indicating that microtubule-dependent compressive forces require cell-substrate adhesion. We also compared three-dimensional (3D) multicellular spheroids with isolated cells and found that ATM, Chk1, and H2AX protein levels were significantly lower in spheroids (Appendix Fig. S5B). Together, these findings strongly suggest that vimentin depletion and mechanical compression act through a common mechanism that remodels chromatin organization and modulates DDR pathways.

### Cell compression and vimentin depletion both promote cell survival following DNA damage

**Figure 7:**
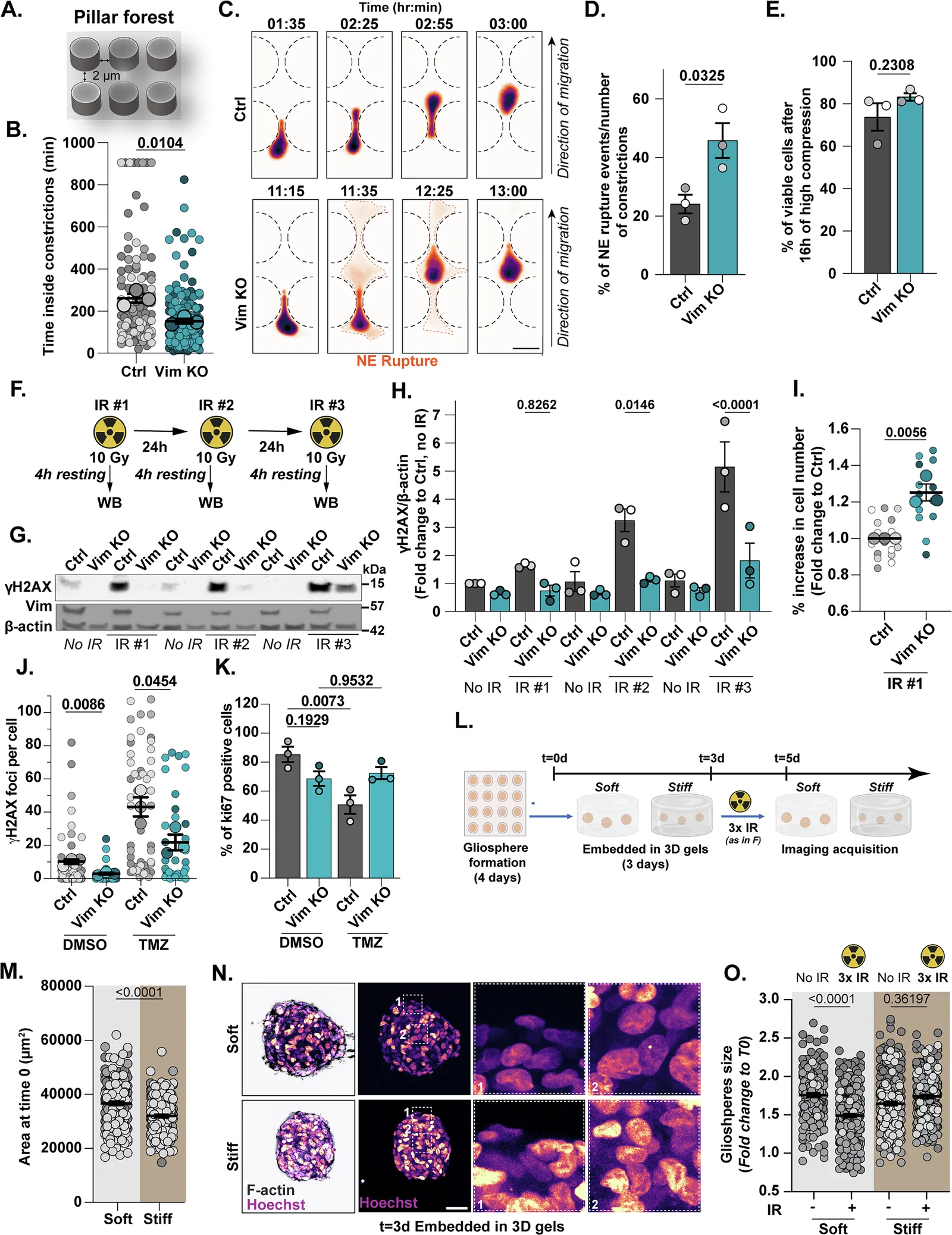
Vimentin loss promotes glioblastoma cell survival after mechanically, physically and chemically-induced DNA damage. (**A**) Schematic representation of the pillar forest microfluidic device. (**B**) SuperPlots of the transition time of control and Vim KO cells through 2 µm-spaced pillars (Movies EV4 and EV5). Individual data points are represented as small dots in grey (Ctrl) or blue (Vim KO). The mean of each independent experiment is indicated by larger dots. Data were acquired from three independent experiments and analysed using a two-tailed *t*-test. Error bars represent mean ± SEM. (**C**) The gallery shows representative non-consecutive frames from time-lapse movies, obtained from three independent experiments of NLS-mCherry control and NLS-mCherry Vimentin KO cells passing through 2-µm-spaced pillars. (Movies EV5 and EV6). (**D**) Quantification of the nuclear envelope rupture events in control and Vim KO cells during passage through 2 µm spaced pillars. Data were acquired from three independent experiments and analysed using a two-tailed *t*-test. Error bars represent mean ± SEM. (**E**) MTT assay of control and Vim KO cells after 16 h of high compression (2 µm). Data were acquired from three independent experiments and analysed by a two-tailed *t*-test. Error bars represent mean ± SEM. (**F**) Schematic representation of different doses of X-Ray irradiation (IR). (**G**,** H**) U251-MG control or Vim KO cells were subjected to different doses of X-rays as indicated, lysed, and the protein level of γH2AX analysed by Western blot. Relative protein expression quantified using Fiji. Data were acquired from three independent experiments and analysed using a one-way ANOVA. Error bars represent mean ± SEM. (**I**) Proliferation of cells was quantified after 72 h of 1 dose of X-Ray irradiation. SuperPlots represent the percentage of increase in cell number. Individual data points are fields of view of Ctrl (grey) or Vim KO (blue). The mean of each repeat is shown by bigger dots. Data were acquired from three independent experiments and analysed by a two-tailed *t*-test. Error bars represent mean ± SEM. (**J**) SuperPlots show γH2AX foci in Ctrl or Vim KO U251-MG treated with DMSO (vehicle) or temozolomide (TMZ, 100 μM, right). Individual data points are shown as small dots in grey (Ctrl) or blue (Vim KO), and the mean of each independent experiment is indicated by larger dots. Data were acquired from three independent experiments and analysed using a two-tailed *t*-test. Error bars represent mean ± SEM. (**K**) Quantification of Ki67-positive ctrl or Vim KO U251-MG cells treated with DMSO or Temozolomide. Data were acquired from three independent experiments and analysed by a one-way ANOVA test. Error bars represent mean ± SEM. (**L**) Experimental flow chart of gliosphere irradiation in a compressive environment. (**M**) Quantification of gliosphere area. Data were acquired from three independent experiments and analysed by a two-tailed t-test. Error bars represent mean ± SEM. (**N**) Representative maximum intensity projection of confocal z-stacks of gliosphere embedded for 3 days in soft or still gel. Insets show zoom of selected area. (**O**) Quantification of gliosphere size after 3 doses of X-Ray irradiation. Data were acquired from three independent experiments and analysed using a mixed linear model. Error bars represent mean ± SEM.

First, we explored the effects of mechanical stresses encountered during invasion through confined environments, by monitoring cell migration through a polydimethylsiloxane (PDMS) microfluidic chip composed of an array of pillars with a 2 µm inter-pillar spacing (Fig. 7A). Vim KO GBM cells traversed constrictions more rapidly than control cells, confirming their increased deformability and enhanced migratory capacity (Fig. 7B; Movies EV3 and EV4). However, the absence of vimentin increased the frequency of nuclear envelope ruptures during migration (Fig. 7C,D and Movies EV5 and EV6). Transient cytoplasmic bursts of a nuclear fluorescent reporter (NLS-mCherry) were twice as frequent in Vim KO cells as in control cells, indicating increased nuclear envelope instability. Interestingly, these experiments also revealed that while the absence of vimentin increases nuclear susceptibility to envelope rupture and potential DNA damage under mechanical stress, it does not affect cell survival (Fig. 7E; Appendix Fig. S5C) in these conditions.

Standard GBM treatment typically involves surgical resection, radiotherapy, and temozolomide chemotherapy (Stupp et al, 2005; Wen and Kesari, 2008). However, the effects of these interventions are often transient, with recurrence and resistance posing major challenges. This resistance is thought to be driven by the profound intratumoural heterogeneity characteristic of GBM (Bhargav et al, 2021; Pichol-Thievend et al, 2024; Qazi et al, 2017). We thus tested the impact of vimentin expression on the cellular response to repeated X-ray exposures (10 Gy per day over three days) (Fig. 7F). γH2AX levels progressively accumulated in control cells following each irradiation, as expected. In contrast, γH2AX levels remained strikingly low in Vim KO cells (Fig. 7G,H). These low levels were not the consequence of a delay, as the kinetics was similar in control and Vim KO cells (Appendix Fig. S5D). Moreover, we observed a higher proliferation rate of Vim KO cells than of control cells following irradiation (Fig. 7I).

The primary chemotherapeutic agent in the treatment of GBM is temozolomide, an alkylating agent which promotes DNA damage. γH2AX levels following treatment (TMZ) were significantly lower in Vim KO cells than in control cells (Fig. 7J; Appendix Fig. S5E). Moreover, while temozolomide treatment induced a strong decrease in proliferative cells in the control situation, it did not affect the proliferation of Vim KO cells (Fig. 7K). This finding suggests that vimentin-negative cells have impaired DNA damage sensing, allowing them to bypass cell cycle checkpoints and evade apoptosis, ultimately enhancing their survival after irradiation.

To determine whether mechanical compression had a similar effect on cell survival following irradiation as vimentin depletion, we used GBM cell gliospheres embedded in non-degradable gels of different rigidity (Fig. 7L). In ‘stiff’ hydrogels (~3 kPa), gliospheres became more compact than when embedded in ‘soft’ hydrogels (~50 Pa) (Appendix Fig. S5F; Fig. 7M), and the cell nuclei appeared significantly smaller (Appendix Fig. S5G), confirming nuclear compression. After 3 days (Fig. 7N), embedded gliospheres were submitted to 10 Gy irradiations every 24 h to mimic therapeutic protocols. Consecutive irradiations significantly reduced gliosphere size in ‘soft’ gels, reflecting decreased cell survival or proliferation, whereas the size of gliospheres embedded in ‘stiff’ gels remained unaffected (Fig. 7O). These observations strongly suggest that cell compression, which, like vimentin loss, represses DDR pathways, enhances cell survival following irradiation.

Our results reveal that the loss of vimentin in both GBM cells and breast adenocarcinoma cells induces nuclear compression, leading to alterations in histone modifications. This nuclear compression, whether caused by vimentin loss or external compressive forces, significantly suppresses DNA damage response (DDR) pathways and DNA damage checkpoints, thereby enabling sustained cell proliferation and survival under stress conditions. These findings highlight a common mechanism by which the absence of vimentin or increased compressive forces allows tumour cells to retain their proliferative capacity despite accumulating DNA damage. In agreement, we observed that Vim KO cells present more micronuclei than control cells (Appendix Fig. S5H), suggesting that over time, the absence of a vimentin-based mechanical shield around the nucleus may promote genomic instability. Altogether, our observation suggests that vimentin-negative cells likely contribute to tumoral genomic instability, intratumoral heterogeneity, and resistance to treatment.

## Discussion

In this study, we demonstrate that both external compressive forces and the loss of vimentin IFs profoundly impact nuclear morphology, chromatin organization, and DNA damage response (DDR) pathways, ultimately enhancing cell survival under physical stresses, including mechanical compression and X-ray irradiation. The ability of mechanical compression to recapitulate the effects of vimentin loss on histone modifications and on the expression of DDR-related genes strongly indicates that the mechanical properties of the vimentin-based IF network are the primary drivers of these changes.

Previous studies, including our own, have shown that vimentin depletion inhibits the expression of other glial IF proteins (GFAP, nestin, synemin), effectively eliminating the IF network from the cytoplasm (van Bodegraven et al, 2025). The absence of vimentin has been associated with increased cellular deformability in various cell types, including normal fibroblasts and cancerous GBM, and breast adenocarcinoma cells, in which it has been linked to nuclear dysmorphia (Pan and Chen, 2024). Consistent with these findings, our results show that vimentin loss significantly reduces the force required to induce large nuclear deformations under compression (Fig. 4I,K). This effect likely arises from the role of the perinuclear vimentin IF cage (Fig. 4A), which acts to limit the transmission of mechanical forces from the cytoplasm to the nucleus (Pan and Chen, 2024; van Bodegraven et al, 2025). Additionally, the contribution of vimentin IFs to the overall elasticity of the cytoplasm may also contribute to their buffering capacity (Infante and Etienne-Manneville, 2022). On a 2D surface, Vim KO cell nuclei experience compressive forces mainly driven by microtubules that accumulate on and around the nucleus in the absence of vimentin IFs. Microtubule-dependent compressive forces require the LINC complex. By contrast, actin makes a minor contribution. We did not detect perinuclear actin accumulation in Vim KO GBM cells, unlike in primary astrocytes (Dupin et al, 2011). Inhibiting ROCK, myosin II, or actin dynamics did not restore nuclear morphology (Appendix Fig. 3E–G). High doses of Cytochalasin D caused cell detachment and rounding, relieving nuclear compression in both WT and Vim KO cells. Accordingly, suspension culture rescued γH2AX levels in vimentin KO cells (Appendix Fig. 5A). Thus, releasing adhesion-dependent mechanical constraints, rather than specifically altering actin architecture, is sufficient to alleviate nuclear compression and associated DNA damage in vimentin-deficient cells. Together, these results indicate that the microtubule-dependent nuclear compression seen in adherent Vim-KO cells requires actin-dependent cell adhesion to the substrate, potentially via microtubule anchoring at focal adhesions (Seetharaman and Etienne-Manneville, 2019). The mechanical protection provided by vimentin IF does not require the LINC complex, suggesting that the steric hindrance caused by the perinuclear cage of vimentin limits the interactions between microtubules and the nucleus. The similar and non-additive effects of vimentin depletion and high compressive forces on histone modifications and DDR gene expression strongly suggest that the vimentin filaments act purely mechanically. Our results indicate that the vimentin network prevents an accumulation of microtubules above the nucleus and microtubule- and LINC complex-dependent nuclear compression. Microtubule-depolymerisation as well as LINC complex inhibition, rescue nuclear shape, as well as histone modification and DDR protein expression. Altogether, these observations suggest that the impact of vimentin loss on gene regulation is mainly due to the mechanical shielding of the nucleus. However, we cannot fully exclude that part of vimentin-dependent transcriptomic regulation may involve biochemical pathways as described for keratin17 (Hobbs et al, 2015), or the direct effect of vimentin as a transcription factor (Fan et al, 2023).

Compressive forces exerted on the cell nucleus, whether through external mechanical stress or the loss of vimentin-mediated resistance, induce nuclear deformation and regulate the epigenetic state through mechanotransduction-dependent pathways (Lv et al, 2018). Epigenetic regulation governs chromatin structure, influencing the balance between transcriptionally inactive heterochromatin and transcriptionally active euchromatin regions (Hu et al, 2022; Uhler and Shivashankar, 2017). These chromatin states are dynamically modulated, and their dysregulation can significantly affect cellular processes such as differentiation, proliferation, and responses to environmental stress or damage (Hu et al, 2022). However, the precise mechanisms by which nuclear compression influences chromatin remodelling enzymes and transcriptional machinery, thereby the resulting gene expression patterns, remain to be elucidated.

Our main result is the fact that nuclear compression, whether it is induced by the loss of vimentin IFs or by external compressive forces or by both, leads to a dramatic downregulation of all major DDR pathways. These pathways include key DNA damage sensors and signalling proteins such as ATM, which activate downstream effectors to halt the cell cycle and facilitate repair or trigger apoptosis if the damage is irreparable. In addition to the downregulation of ATM, we observe a strong inhibition of H2AX. H2AX phosphorylation plays a pivotal role in the DNA damage response, particularly in the repair of double-strand breaks (DSBs). Its phosphorylation at serine 139 by ATM, ATR, or DNA-PK to form γH2AX acts as a molecular beacon to amplify the DDR signal, recruiting and retaining DNA repair proteins at the damage site. Here, not only are the major DDR kinases downregulated, but H2AX expression is also strongly reduced, leading to a dramatic decrease in γH2AX, even when DNA damage is strongly induced by UV or X-Rays (Figs. 3 and 6, respectively). In addition to the alteration of DNA damage sensing, DNA repair proteins, including 53BP1, are also downregulated. Decreased expression and activation of these tumour suppressors, which normally ensure the stability of the genome, likely lead to accumulation of genomic alterations (Gorodetska et al, 2019; Gudmundsdottir and Ashworth, 2006). Finally, PARP1, which is directly involved in the repair of single-strand DNA breaks to prevent these lesions from converting into more dangerous double-strand breaks during DNA replication, is also downregulated, likely also contributing to genomic instability (Ray Chaudhuri and Nussenzweig, 2017). Our results show that GBM cells that lack vimentin or are submitted to large compressive forces lack most of the DNA damage-sensing pathways and thus cannot efficiently repair DNA, leading to persistent DNA lesions. Accumulation of such damage increases genomic instability, which can drive tumour progression and promote the emergence of cancer subclones with adaptive advantages. This may contribute to the effect of mechanotransduction on glioblastoma growth and progression (Khoonkari et al, 2022).

Defects in double-strand DNA break repair should activate cell cycle checkpoints or apoptosis pathways as a protective mechanism. However, when exposed to UV, X-Rays or constraint-induced nuclear envelope leakage, Vim KO cells proliferate more and undergo less apoptosis than control cells. This surprising observation can be explained by the downregulation of major targets of the DDR pathway, which should mediate cell cycle checkpoints. For instance, loss of vimentin decreases the serine/threonine kinase Chk1 (Figs. 1 and 2), which phosphorylates and inhibits CDC25 to induce cell cycle arrest (Uto et al, 2004). Moreover, 84% of GBM patients and 94% of GBM cell lines, including U251-MG cells, display altered p53-ARF-MDM2 pathway (Zhang et al, 2018) and low expression of vimentin has been correlated with a decrease in p53 (Sembritzki et al, 2002), suggesting that the p53 pathway is generally attenuated in vimentin-negative GBM cells. Finally, apoptosis is also diminished in Vim KO cells. Although we did not detect any major changes in the level of expression of Bcl2 family members, there was an inhibition of APAF-1, another major player of the apoptotic intrinsic pathway (Fig. 1E). Inhibition of APAF-1 not only prevents procaspase-9 activation and apoptosis, but it also prevents mitochondrial damage, which may also contribute to the cell survival that we observed following UV irradiation exposure (Fig. 3C–K) (Gortat et al, 2015). We propose that the profound alteration of the DDR pathways, together with a downregulation of the downstream signalling normally leading to cell cycle arrest or cell death, leads to the accumulation of DNA damage while the cell keeps proliferating. Ultimately, this will likely result in some level of cell death, but it may also strongly contribute to intratumoral heterogeneity.

In GBM, cancer cells are softer than healthy astrocytes. Notably, grade IV GBM cells, the most invasive, are softer than grade III astrocytoma cells (Alibert et al, 2021). While these cells are softer overall due to reduced cytoskeletal protein levels, they exhibit increased perinuclear stiffness due to the local accumulation of actin and IFs. The lower expression of vimentin within the tumour core compared to the periphery (Fig. 1B) suggests that this region may be prone to the accumulation of mutations, whereas the higher proportion of vimentin-positive cells at the tumour periphery would promote invasion. However, we previously showed that vimentin-positive cells could lead to the migration of vimentin-negative cells (Pan and Chen, 2024; van Bodegraven et al, 2025). This suggests that vimentin-negative cells may also disseminate and contribute to tumour recurrence.

Importantly, cell compression can trigger similar effects as the loss of vimentin. We did not detect any changes in vimentin expression level following overnight compression, but we cannot exclude that in the long term, such regulatory mechanisms may also be at play. As GBM grows within the brain tissue, it exerts a local pressure on its surroundings and results in a tissue displacement known as the gross mass effect, responsible for the acute neurological symptoms seen in patients. However, because the space occupied by the brain is restricted by the cranium, tumour growth also implies compression of the tissue (Ropper, 1986). We show that gliospheres growing within a stiffer environment (thus, a more limited space) have better resistance against X-Ray exposure (Fig. 6O), suggesting that this mechanical compression could also promote tumour radioresistance.

Since loss of vimentin also leads to the alteration of nuclear shape, of chromatin marks and to the downregulation of DDR pathways in GBM and breast adenocarcinoma cells (Appendix Figs. 2G,H; Fig. 5D), we propose that the mechanisms described here may be at play in several cancer types (Pan and Chen, 2024). Altogether, these findings underscore the pivotal role of cancer mechanics in disease progression (Linke et al, 2024; Mohammadi and Sahai, 2018). Both the mechanical properties of the tumour microenvironment and of the tumour cells themselves significantly influence tumour cell resistance to DNA damage, probably contributing to radiotherapy resistance and promoting tumour heterogeneity. These mechanical factors also likely impact tumour responses to chemotherapies targeting DNA damage response (DDR) pathways, as softer or compressed cells can evade apoptosis and continue proliferating despite impaired DNA repair mechanisms.

Moreover, our results highlight vimentin as a key player in tumour progression. While vimentin expression facilitates tumour cell invasion, its absence paradoxically enhances tumour cell resistance to therapy and promotes genomic alterations. These findings suggest that vimentin expression could serve as a valuable molecular marker to differentiate tumour cells with distinct behaviours, potentially guiding the selection of the most effective treatment strategies. This work paves the way for further exploration of vimentin as both a diagnostic tool and a therapeutic target in cancer management.

### Limitations of the study

Because of the genetic and phenotypic diversity of glioblastoma cells, drawing conclusions on glioblastoma cell biology is a challenge. We have sought without success a correlation between vimentin and DDR gene expression in single cell and spatial transcriptomics. However, the fact that the mechanical environment (in particular, compression) of cells, which is not known in these transcriptomic analyses, can lead to a similar phenotype as the absence of vimentin, does not allow us to draw any clear conclusions. Moreover, most of the study is performed on a classically used patient-derived glioblastoma cell line, U251-MG, whose responses to DNA damage or vimentin loss can only be cautiously generalised to all glioblastoma cells. Nevertheless, we did observe a similar role of vimentin in the regulation of DDR pathways in a more recently purified patient-derived cell line, U3117 from Uppsala, and also on MDA-MB-231 adenocarcinoma cell lines. These converging evidence support the idea that vimentin expression, while shown to increase cancer cell invasion, may also diminish the resilience of these cells and that vimentin-negative, probably less invasive cells, may instead be crucial for tumour resistance to treatment and tumoral heterogeneity.

## Methods

**Table:** Reagents and tools table

Antibodies	Source	Reference
Mouse monoclonal anti-Vimentin	Santa Cruz Biotechnology	sc-6260
Mouse monoclonal anti-β-actin	Sigma-Aldrich	A2228
Mouse monoclonal anti-GAPDH	Merck	MAB374
Rat monoclonal anti-a-Tubulin	Bio-Rad	MCA77G
Rabbit polyclonal anti-H2AX	Cell signaling	2595
Rabbit polyclonal anti-pH2AX (S139)	Cell signaling	2577
Mouse monoclonal anti-pH2AX	Merck	JBW301
Rabbit polyclonal anti-ki67	Abcam	15580
Rabbit monoclonal anti-ATM	Cell signaling	2873
Mouse monoclonal anti-lamin A/C	Santa Cruz Biotechnology	376248
Rabbit monoclonal anti-H3me3K27	Cell signaling	9733
Rabbit monoclonal Acetyl-Histone H3 (Lys9)	Cell signaling	9643
Mouse monoclonal Histone H3	Cell signaling	14269
Rabbit monoclonal anti-H3me3K9	Cell signaling	13969
Mouse monoclonal anti-PCNA	Cell signaling	2586
Rabbit monoclonal anti- NF-kB	Cell signaling	8242
Rabbit polyclonal anti-SUN1	Sigma-Aldrich	HPA008461
Mouse monoclonal anti-Chk1	Cell signaling	2360
Rabbit polyclonal anti-Chk1	Bethyl laboratory	300-298 A
HRP- Donkey Anti-rabbit	Jackson ImmunoResearch	711-035-152
HRP- Donkey Anti-Mouse	Jackson ImmunoResearch	715-035-150
Tetramethylrhodamine (TRITC) donkey anti-rabbit		(711-025-152)
Alexa Fluor 488 donkey anti-mouse	Jackson ImmunoResearch	715-545-151
Anti-actin hFAB Rhodamine antibody	Bio-Rad	12004163
Alexa Fluor 647 phalloidin	Thermo Fisher Scientific	A22287
Annexin V-FITC	Abcam	Ab14085
**siRNA**	**Source**	**Sequence**
siCtrl	Eurofins	UAAGGCUAUGAAGAGAUAC
siVim#1	Eurofins	UAAGCUCUCUAGUUCUUAACA
siVim#2	Eurofins	UGAAGAAACUCCACGAAGA
siLMNA#1	Eurofins	AGAAGGAGCUGGAGAAGAC
siLMNA#2	Eurofins	GGAUGAGGAUGGAGAUGAC

### Cell culture, stable cell lines, siRNA and drug treatments

U251-MG cells were grown in Minimum Essential Medium (MEM) with GlutaMAX supplement (Thermo Fisher Scientific) supplemented with 10 mM of MEM non-essential amino acid, 100 U ml^−1^ penicillin, 100 mg ml^−1^ streptomycin (Gibco) and 10% foetal calf serum (Eurobio Scientific) at 37 °C under 5% CO2. U3117 cells were grown on Geltrex (Gibco) pre-coated plates and grown in serum-free GBM complete medium composed of Neurobasal-A medium mixed 1:1 (v/v) with DMEM/F12 + GlutaMAX and complemented with human FGF-basic and human EGF at 20 ng/mL, and B-27 (Gibco).

Vimentin KO U251-MG cell line was generated as described before (Jiu et al, 2017). Guide RNAs (sgRNAs) to target Vimentin as described in Jiu et al, 2015, with high on-/off-target ratios for different exons were used. The pSpCas9(BB)-2A-GFP plasmid (PX458, Addgene, Catalogue #48138) containing a gRNA scaffold under the U6 promoter and Cas9 and EGFP under the CMV promoter was linearised with BbsI-HF (NEB) and specifically designed annealed oligonucleotides containing the guide sequences (sgRNA 1, targeting exon 2: 5’ CACCGTGGACGTAGTCACGTAGCTC-3’ and 5’- AAACGAGCTACGTGACTACGTCCAC-3’, sgRNA 2, targeting exon 2: 5’- CACCGCAACGACAAAGCCCGCGTCG -3’ and 5’- AAACCGACGCGGGCTTTGTCGTTGC -3’) were ligated into the linearised plasmid using T4 Ligase (Thermo Fisher Scientific).

Patient-derived U3117 GBM cells were acquired from the Human Glioblastoma Cell Culture resource (http://www.hgcc.se) at the Dept. of Immunology, Genetics and Pathology, Uppsala University, Uppsala, Sweden (Xie et al, 2015). Cells were transfected with the sgRNA-containing plasmids by Lipofectamine 2000 (Thermo Fisher Scientific) according to the manufacturer’s protocol. Cells were plated in culture dishes and maintained for three days. GFP-positive cells were subsequently collected using fluorescent activated cell sorting (FACS) and plated as single cells in 96-well plates, and were grown till confluency and maintained. Vimentin knockout was determined using immunostaining and western blot analysis. sgRNA 2 resulted in the highest number of vimentin KO clonal lines. A control and KO clonal line were selected based on the absence of vimentin protein and an IF network and were used for analyses.

To generate stable U251 cell lines overexpressing or re-expressing vimentin (in Vim KO cells), cells were transduced with lentiviral constructs pLKO-hVimentin-puro for 24 h. Forty-eight hours after transduction, cells were selected by treatment with 1 µg/ml puromycin. Vimentin was tested by Western blot. Reconstitution of an IF network upon re-expression of vimentin was confirmed by immunostaining (van Bodegraven et al, 2025).

U251-MG cells stably expressing NLS-mCherry were generated by nucleofection (Amaxa Biosystems Nucleofection System) with 1 µg of DNA in 100 µl of Glial nucleofection reagent (Lonza Biologics), followed by selection with 500 µg ml^−1^ geneticin (Thermo Fisher Scientific). After fluorescence-activated cell sorting (FACS), polyclonal cell lines were obtained and cultured in complete media supplemented with 500 µg ml^−1^ geneticin.

U251-MG cells stably expressing KASHext or DN-KASH were generated by nucleofection (Amaxa Biosystems Nucleofection System) with 1 µg of DNA in 100 µl of Glial nucleofection reagent (Lonza Biologics), followed by selection with 500 µg ml^−1^ geneticin (Thermo Fisher Scientific). After fluorescence-activated cell sorting (FACS), polyclonal cell lines were obtained and cultured in complete media supplemented with 500 µg ml^−1^ geneticin.

Vimentin-rescued U251-MG cell lines were generated by re-expression of vimentin cDNA in vimentin-depleted cells from Vimentin-7-EGFP (Addgene #56439), modified by introducing two stop codons between the vimentin sequence and the EGFP reporter sequence. Cells were transfected using Lipofectamine 2000 (Thermo Fisher Scientific) according to the manufacturer’s protocol. Three days post-transfection, cells were treated with 500 µg ml^−1^ geneticin to select transfected cells. A single cell clone was generated and selected based on immunoreactivity for vimentin.

MDA-MB-231 were grown in DMEM-High Glu medium supplemented with 100 U ml^−1^ penicillin, 100 mg ml^−1^ streptomycin and 10% foetal calf serum at 37 °C under 5% CO_2_.

U251-MG and MDA-MB-231 cells were transfected by nucleofection using 1 nmol siRNA (Sigma-Aldrich) in 100 μl of Nucleofection reagent (kit C, Lonza Biologics, Slough, UK). Experiments were carried out 72 h post-transfection. siRNA sequences used in this study are listed as follows: Luciferase (siCtrl)-UAAGGCUAUGAAGAGAUAC; siVim#1-UAAGCUCUCUAGUUCUUAACA; siVim#2-UGAAGAAACUCCACGAAGA; siLMNA#1-AGAAGGAGCUGGAGAAGAC; siLMNA#2-GGAUGAGGAUGGAGAUGAC.

Cells were treated with 10 μM Nocodazole, 5 μM RockI and II inhibitor GSK269962A or 5 μM Y27632, 100 μM temozolomide, cytochalasin D (100 or 200 nM) or Blebbistatin (200 nM or 2 μM).

All cell lines were tested for mycoplasma.

### Analysis of GBM patient-derived published single-cell RNA sequencing data

To explore vimentin expression in patient-derived GBM samples, a large integrated GBM single-cell RNA sequencing dataset, GBM Map24, was used. The Seurat object containing integrated and annotated data was downloaded (Data ref: Ruiz-Moreno, 2022) and used for analyses using Seurat 5.0.1 (Hao et al, 2021). Samples from recurrent GBM were excluded from analyses. To explore vimentin expression in core- and periphery-derived samples, single-cell RNA sequencing data from Darmanis et al, 2017 (Data ref) was used and processed using Seurat 5.0.1. To determine the percentage of vimentin-positive and negative cells per patient, raw read counts were used. Cells with >1 raw count for vimentin were annotated as vimentin-positive cells, while cells with <1 raw count for vimentin were annotated as cells in which vimentin was not detected (negative cells).

### RNA sequencing

RNA from cells was extracted on ice using a RNeasy Mini kit (Qiagen) according to the manufacturer’s instructions and treated with DNase. RNA concentration was determined by measuring the ratio of the absorbances at 260 and 280 nm. Quality control of RNA samples was assessed with a 2100 Bioanalyser system according to the manufacturer’s instructions.

Libraries were prepared from 300 ng of RNA using Illumina Stranded mRNA Prep (Illumina) following the manufacturer’s protocol. Briefly, mRNA was captured by oligo-dT magnetic beads, fragmented and reverse transcribed. Sample-specific barcodes were ligated to the cDNA and then amplified by 17 cycles of PCR. Note that only the first cDNA strand was amplified. Purification of unbound adaptors and primers was done on AMP beads (Beckman Coulter) added to samples in a 0,8:1 ratio. The resulting stranded libraries comprised fragments from 200 to 1000 bp with a peak around 490 bp, as visualised on a 5300 Fragment Analyser (Agilent Technologies). Libraries were pooled, purified on AMP beads as described above, diluted to 0.65 nM and sequenced on a NextSeq 2000 system (Illumina) using a P3 50-cycle sequencing kit. The yield was 1100 mln 67- bp single-end reads (30 – 60 mln reads per sample).

### RNA-seq and statistical analysis

The RNA-seq analysis was performed with Sequana 0.14.1 (Cokelaer et al 2017). We used the RNA-seq pipeline 0.18.1 (https://github.com/sequana/sequana_rnaseq) built on top of Snakemake 7.8.5 (Köster and Rahmann 2012). Briefly, reads were trimmed from adaptors using Fastp 0.20.1 (Chen et al, 2018), then mapped to the Homo sapiens GRCh38 genome assembly from Ensembl using STAR 2.7.8a (Dobin et al, 2013). FeatureCounts 2.0.1 (Liao et al, 2014) was used to produce the count matrix, assigning reads to features using corresponding annotation v94 from Ensembl with strand-specificity information.

RNA-seq count data were analysed using R version 4.2.1 (R, 2024) and the Bioconductor package DESeq2 version 1.36.0 (Love, 2024). The normalisation and dispersion estimation were performed with DESeq2 using the default parameters, and statistical tests for differential expression were performed by applying the independent filtering algorithm. A generalised linear model was set in order to test for the differential expression between VIM and control. Raw *p* values were adjusted for multiple testing according to the Benjamini and Hochberg (BH) procedure (Hochberg, 1995), and genes with an adjusted *p* value lower than 0.05 were considered differentially expressed. Gene set enrichment analysis was performed using the fgsea R package (preprint: Korotkevich et al, 2019; Subramanian et al, 2005) on the Wikipathways gene-sets (Agrawal et al, 2024). Only gene sets with a FDR lower than 0.05 were considered significantly enriched.

### Western blot analysis

Cells were lysed using Laemmli buffer with protease and phosphatase inhibitor cocktails. Lysates were separated by SDS–PAGE and transferred to a polyvinylidene difluoride membrane. Membranes were incubated in blocking buffer (3% BSA in PBS containing 0.1% Tween-20) for 1 h at room temperature (RT) and then incubated overnight at 4 °C with the indicated antibodies. Antibodies were visualised using either the ECL detection system or fluorescent secondary antibodies (Bio-Rad). Signals were recorded using a ChemiDoc MP Imaging System (Bio-Rad). Densitometric analysis was performed using FIJI.

### Immunofluorescence microscopy

Samples were fixed with 4% PFA at RT for 10 min or, for microtubule staining, with cold methanol at −20 °C for 10 min. Samples were then washed three times with PBS 1x. Blocking was performed using a 5% BSA in PBS 1x solution for 1 h at RT. The primary antibodies diluted in 5% BSA in PBS 1x were then incubated 1 h at RT. After three washes using 5% BSA in PBS 1x, samples were incubated with secondary antibodies for 1 h at RT. Samples were then washed three times in PBS 1x1x, and mounted with ProLong Gold antifade reagent with DAPI. Images were acquired either by epifluorescence microscopy using a Leica DM6000 microscope equipped with a 40×1.25 NA or 63×1.4 NA objective or using a LSM 700 confocal microscope with a plan-Apochromat 40X/1.4 Oil objective.

### Nuclear volume quantification

Nuclear volume analysis was performed using a custom FIJI macro. The macro processes TIFF images by segmenting the nuclei through thresholding and image binarisation, ensuring accurate delineation of nuclear boundaries. After segmentation, 3D reconstruction of the nuclei is performed based on the image stack to account for volumetric data. Their volumes are then calculated based on predefined input parameters, with the results saved in a designated output folder.

### Heterochromatin quantification

DAPI-stained images were analysed with Fiji. First, nuclei were segmented to define ROIs, and DAPI intensities were measured for each ROI. High-intensity chromatin regions were thresholded to calculate the percentage of heterochromatin per nucleus.

### γH2AX foci quantification

Nuclei were first identified from DAPI images by segmentation and used to define nuclear ROIs. For each nuclear ROI, the background signal was subtracted from the corresponding γH2AX channel image, and the γH2AX image was thresholded to create a binary mask of foci. Within each nuclear ROI, γH2AX foci were quantified using the Analyze Particles tool with consistent size and circularity settings across all images to count foci per nucleus.

### Cell compression

Cells were plated on a six-well plate in glass-bottom dishes (MatTek, P06G-1.5-20-F) 16 h prior to confinement using a commercial confiner (4D cell, France). Confiner slides were generated internally. The template for the slides was fabricated by soft photolithography according to the protocol previously described (Heuze et al, 2011). To prepare the slides, a drop of polydimethylsiloxane mixture (PDMS RTV 615A and cross-linker B at a ratio of 10:1 (w/w), Neyco, France) was placed on the mould that contained the pillars of target height (~3.5 or ~2 µm) and immediately covered with a 12-mm glass coverslip. The wafers were baked at 95 °C for 15 min and coverslips covered with PDMS pillars were carefully removed using isopropanol. After washing them with water and letting them dry, coverslips covered with PDMS pillars were placed on the soft spacer used to link the lid of the dish to the bottom of the well and incubated in cell medium for 2 h before confinement. Cells were either imaged by time-lapse microscopy or lysed after 4 or 16 h of compression. Migration speed (μm/min) was obtained by tracking cells using the Manual Tracking Fiji analysis software plugin.

### Pillar forest microfluidic device

Pillar forests were prepared from moulds fabricated in the same manner as the ones used in the compression experiments (see Cell compression section in Materials and Methods). The microfluidic chip was prepared by casting 5 g of a mixture of PDMS RTV615 (Neyco, France) at a ratio 10:1 w/w PDMS A:cross-linker B. The mixture was then degassed under vacuum and cured at 65 °C for 1 h. The chip was then removed from the mould and resized to fit the glass part of a glass-bottom 35 mm dish (Fluorodish FD-035, WPI) and 2.5 mm holes were punched out (Biopunch, TedPella) in order to inject cells into the chip. After sonication of the PDMS chip in 70% ethanol, the glass and the chip were air-plasma treated for 30 s at max power (Harrick PDC-002). Both activated surfaces were then put in contact to ensure sticking. To strengthen the bonding, the dish containing the chip was set in an oven at 65 °C for 1. Finally, the chip was washed with PBS and incubated with complete medium for at least 1 h before adding 100,000 cells to each entry.

### X-Ray and UV irradiation

Cells or spheroids were irradiated using an X-Ray generator (Faxitron) at the indicated doses or with an UV (312 nm) irradiation system (UVITEC, Cambridge).

### Comet assay

Comet assay was performed following the manufacturer's instructions (Abcam, ab238544). Briefly, agarose drops were placed on a comet slide to form a base layer. Subsequently, cells subjected to UV irradiation were mixed with agarose and seeded on top of the base layer. Following polymerisation at 4 °C, cells were lysed, and the slides were placed in an electrophoresis tank in alkaline solution. After electrophoresis, samples were stained using a DNA dye and imaged with DMI6000B Leica epifluorescence inverted microscope equipped with10X objective and a Photometrics MOMENT sCMOS camera controlled by Micromanager (Edelstein et al, 2014).

### MTT assay

Cells were plated overnight on a 96-well plate. Cells were exposed to either UV or high compression forces, and after 16 h, tetrazolium salt was added according to the manufacturer’s instructions (Roche). Optical density was measured at 570 nm with the Safas M96 plate reader and analysed with Safas software.

### Live-cell imaging

Time-lapse images were acquired with 10X or 20X objective using a Nikon Eclipse Ti-E epifluorescence inverted microscope with an EMCCD camera/pco.edge sCMOS camera and Metamorph software. Cells were maintained under 5% CO_2_ at 37 °C. Images were acquired every 5 or 15 min for 16 h according to the experimental setup. Cells were manually tracked with Fiji software (Manual Tracking plug-in).

### HoloMonitor

About 1 × 10^4^ cells were plated on a 12-well plate (Sarstedt), and holographic images were acquired for 7 days according to the HoloMonitor cell proliferation protocol. Five positions of two separate wells were acquired per condition. Automated quantification was obtained using the HoloMonitor App Suite.

### Generation of spheroids

Spheroids were prepared using a MicroTissues silicone mould (12-256 - Small Spheroids, MicroTissues). About 2% molten agarose was poured into the silicone mould and allowed to cool in the refrigerator for 5 min. The agarose cuvettes were then demolded into a six-well plate and soaked in PBS 1X supplemented with 1% penicillin-streptomycin and stored at fridge temperature at 4 °C until use. The agarose cuvettes were placed in an incubator before the dissociation of the cells to bring them to 37 °C.

The PBS 1X solution was aspirated just before depositing the cell solution. The spheroids were generated by pipetting 150 µL of dissociated cell suspensions in agarose cuvettes at a concentration of 106 cells/mL. Then, complete GBM medium was added, and the spheroids were left to grow for 4 days under 5% CO_2_ at 37 °C. After 4 days, the generated spheroids were gently flushed out of the cuvette with medium and collected in a Falcon tube. They were then centrifuged at 1000 RPM for 3 min to pellet them down. The supernatant was gently removed, and 200 µL of fresh medium was then added to resuspend the spheroids.

### Spheroids encapsulation in hydrogel

The hydrogel solution was prepared following the protocol provided by Cellendes. Two types of hydrogel were generated: the 'soft' hydrogel, using the fast-gelling dextran hydrogel at a final concentration of 1.5 mM (FG90-1, Cellendes GmbH), and the 'stiff' hydrogel, using the slow-gelling dextran hydrogel at a final concentration of 3 mM (G92-1, Cellendes GmbH). The solutions were left to polymerise at 37 °C for 10 or 15 min, respectively, before the addition of fresh medium. The encapsulated spheroids were then left to grow for 2 days before the first series of irradiation.

### Statistical analysis

Graphs were generated using GraphPad Prism (10.2.1). Statistical tests used in this study is indicated in each figure legend.

No blinding was performed during the study.

## Supplementary information

Appendix

Peer Review File

Movie EV1

Movie EV2

Movie EV4

Movie EV3

Movie EV6

Movie EV5

## Data Availability

The single-cell RNA sequencing data from Ruiz-Moreno et al, 2022 and Darmanis et al, 2017 are available at https://zenodo.org/records/6962901 and GSE84465, respectively. The raw and processed RNA sequencing data of U251 WT and Vim KO cells are available on GEO under accession number GSE286944 (https://www.ncbi.nlm.nih.gov/geo/query/acc.cgi?acc=GSE286944) using the following password: svmvuqmytvsbxyp. The source data are available in the following database record: https://www.ebi.ac.uk/biostudies/studies/S-BSST3076. The source data of this paper are collected in the following database record: biostudies:S-SCDT-10_1038-S44319-026-00913-2.
