# Supplementary material for: Vimentin loss inhibits DNA damage responses and promotes cancer cell survival
Source: EMBO Rep. 2026 Sep 4;27(19):5561–81. doi: 10.1038/s44319-026-00913-2 (PMC13649785; doi:10.1038/s44319-026-00913-2)
Supplement: Supplementary file 1 [file 44319_2026_913_MOESM1_ESM.pdf]

Appendix

Table of contents

Appendix Figure S1……………………………………………………………..….…………2
Appendix Figure S2……………………………………………………………..…….………3
Appendix Figure S3………………………………………………………….……..……… 5
Appendix Figure S4……………………………………………………….……..…………..… 7
Appendix Figure S5…………………………………………………………………………9

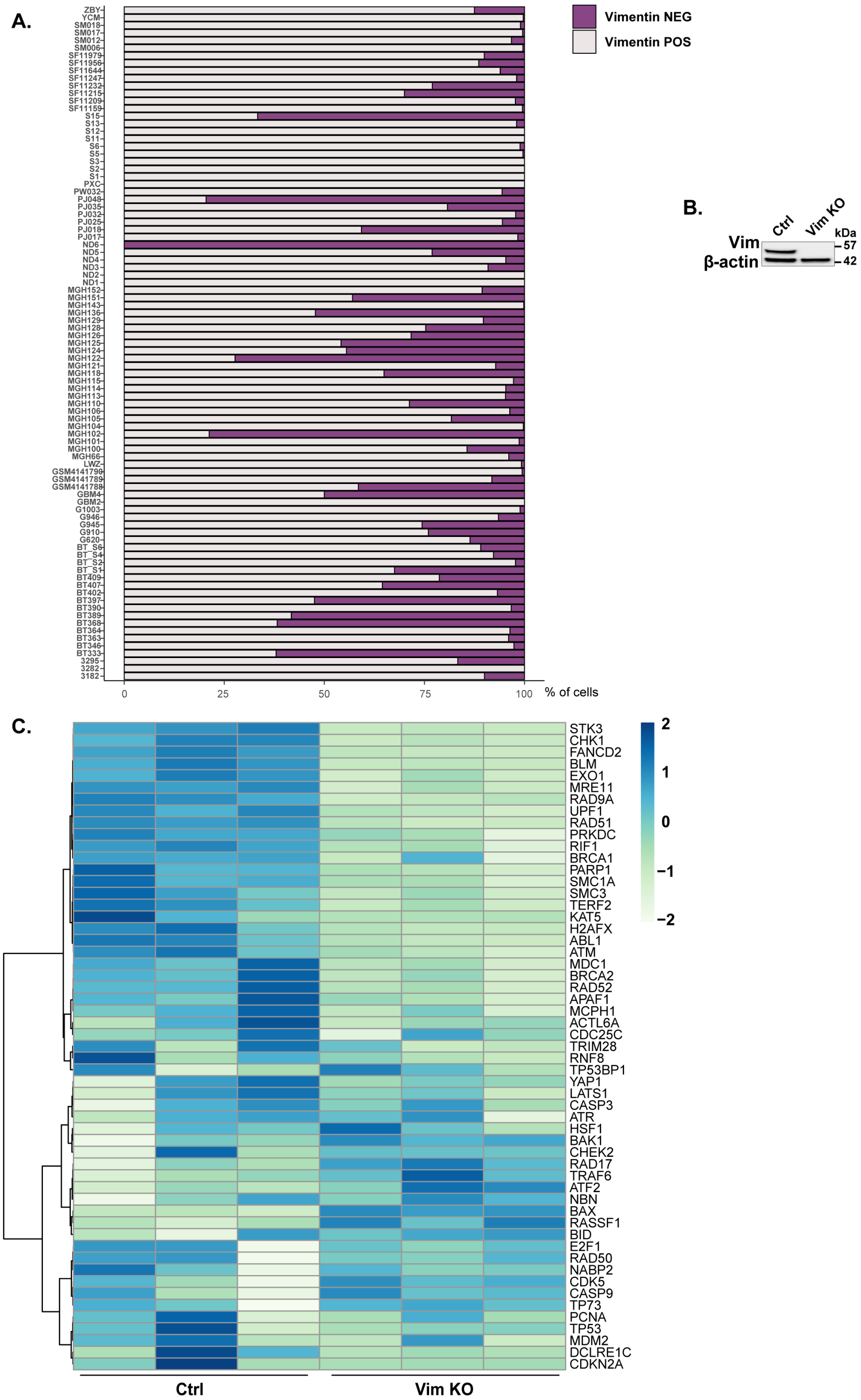

Appendix Figure S1.

A. Percentage of vimentin-positive cells per patients from single cell RNA sequencing dataset.

B. Expression of vimentin protein in control and Vim KO U251-MG cells was analysed by western blot.

C. Heatmaps providing a full visual representation of gene expression of the DNA IR-double strand breaks and cellular response via ATM pathways in control versus Vim KO cells.

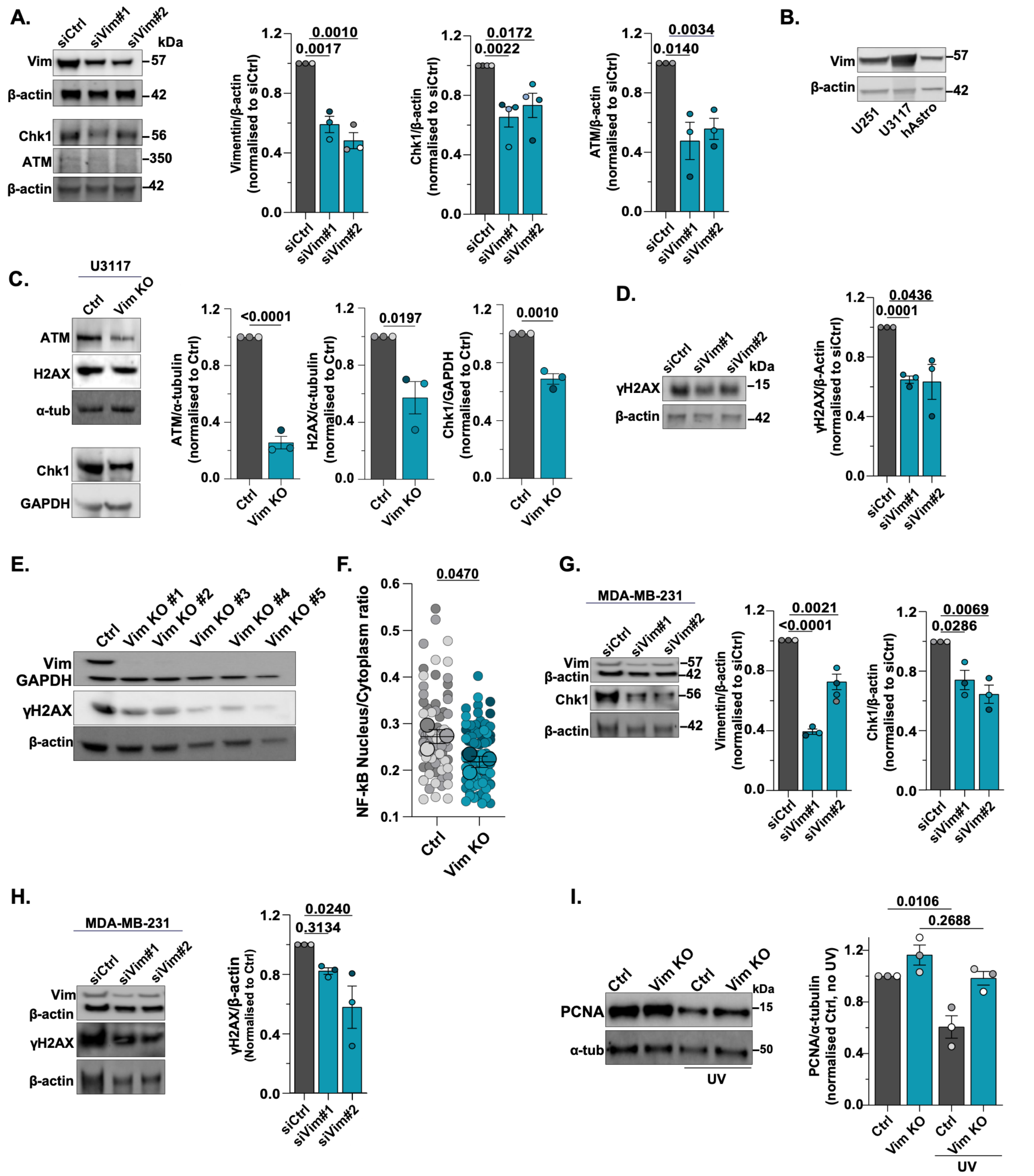

Appendix Figure S2.

A. U251-MG cells were transfected with siRNAs targeting vimentin. Levels of vimentin, Chk1, and ATM proteins were analysed by western blot, and relative expression was quantified using Fiji. Data were acquired from three independent experiments (four for Chk1) and analysed by one-way ANOVA. Error bars represent mean ± SEM.

B. Expression of vimentin in U251-MG, U3117 primary cell line and human astrocytes.

C. Chk1, ATM, and H2AX expression in control and Vim KO U3117 primary cells were analysed by western blot, and relative expression was quantified using Fiji. Data were

acquired from three independent experiments and analysed by two-tailed *t*-test. Error bars represent mean ± SEM.

D. Vimentin expression was downregulated in U251-MG cells by siRNA, and γH2AX expression was analysed by western blot. Relative expression was quantified using Fiji. Data were acquired from three independent experiments and analysed by a two-tailed *t*-test. Error bars represent mean ± SEM.

E. Expression of γH2AX in control and five single clones of Vim KO U251-MG cells analysed by western blot.

F. SuperPlots of nucleus-to-cytoplasm ratio of NF-κB measured by immunofluorescence. Individual data points are shown as small dots in grey (Ctrl) or blue (Vim KO); the mean of each repeat is shown as larger dots. Data were acquired from three independent experiments and analysed by two-tailed *t*-test. Error bars represent mean ± SEM.

G. Expression of Chk1 in MDA-MB-231 cells depleted or not for vimentin by siRNA was analysed by western blot, and relative expression was quantified using Fiji. Data were acquired from three independent experiments and analysed by two-tailed *t*-test. Error bars represent mean ± SEM.

H. Expression of γH2AX in MDA-MB-231 cells depleted or not for vimentin by siRNA was analysed by western blot, and relative expression was quantified using Fiji. Data were acquired from three independent experiments and analysed by one-way ANOVA. Error bars represent mean ± SEM.

I. U251 cells depleted or not for vimentin were irradiated with 210 mJ/cm² UV light, lysed after 24h, and PCNA protein levels were analysed by western blot and quantified using Fiji. Data were acquired from three independent experiments and analysed by one-way ANOVA. Error bars represent mean ± SEM.

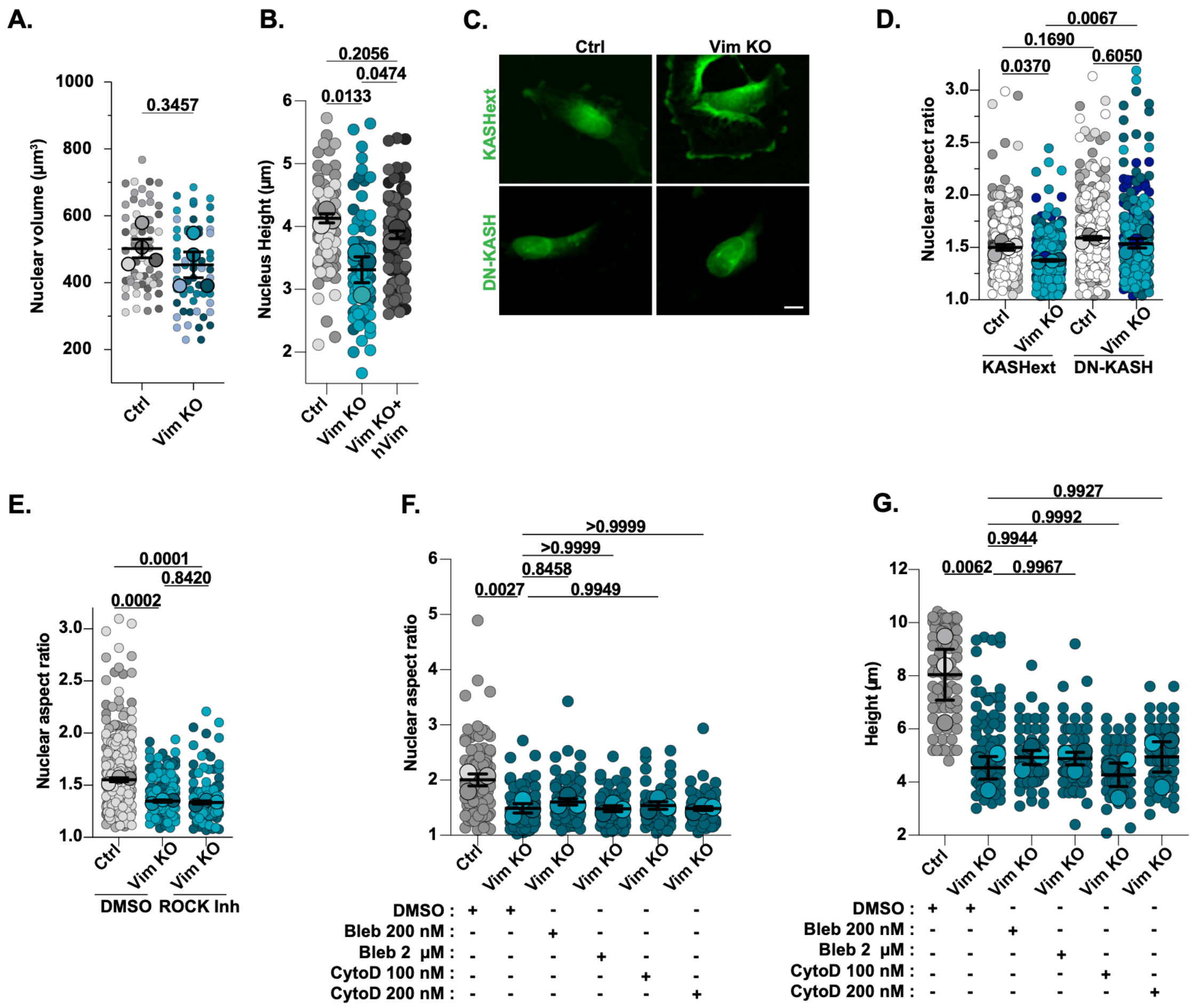

Appendix Figure S3.

A. SuperPlots of nuclear volume. Individual data points are shown as small dots in grey (Ctrl) or blue (Vim KO); the mean of each repeat is shown as larger dots. Data were acquired from three independent experiments and analysed by two-tailed *t*-test. Error bars represent mean ± SEM.

B. SuperPlots of nuclear height. Individual data points are shown as small dots in grey (Ctrl), blue (Vim KO) or dark grey (Vim KO + human Vim); larger dots indicate the mean of each independent experiment**.** Data were acquired from three independent experiments and analysed by one-way ANOVA*.* Error bars represent mean ± SEM.

C. Representative images of Ctrl or Vim KO U251-MG cells stably expressing KASHext or DN-KASH constructs (scale bar 10 µm).

D. SuperPlots of nuclear aspect ratio in Ctrl or Vim KO U251-MG cells stably expressing KASHext (left) or DN-KASH (right). Individual data points are shown as small dots in grey (Ctrl) or blue (Vim KO); the mean of each repeat is indicated by larger dots. Data were acquired from three independent experiments and analysed by one-way ANOVA. Error bars represent mean ± SEM.

E. SuperPlots of nuclear aspect ratio of U251-MG cells treated either with either DMSO (vehicle) or ROCK inhibitor (5 μM GSK269962A for 24h). Individual data points are shown as small dots in grey (Ctrl) or blue (Vim KO); the mean of each repeat is symbolized by bigger dots. Data were acquired from three independent experiments and analysed by one-way ANOVA. Error bars represent mean ± SEM.

F. SuperPlots of nuclear aspect ratio of U251-MG cells treated either with either DMSO (vehicle) or Blebbistatin (200 nM or 2 μM) or cytochalasin D (100 nM or 200 nM) for 24h. Individual data points are shown as small dots in grey (Ctrl) or blue (Vim KO); the mean of each repeat is symbolized by bigger dots. Data were acquired from three independent experiments and analysed by one-way ANOVA. Error bars represent mean ± SEM.

G. SuperPlots of nuclear height of U251-MG cells treated either with DMSO (vehicle) or Blebbistatin (200 nM or 2 μM) or cytochalasin D (100 nM or 200 nM) for 24h. Individual data points are shown as small dots in grey (Ctrl) or blue (Vim KO); the mean of each repeat is symbolized by bigger dots. Data were acquired from three independent experiments and analysed by one-way ANOVA. Error bars represent mean ± SEM.

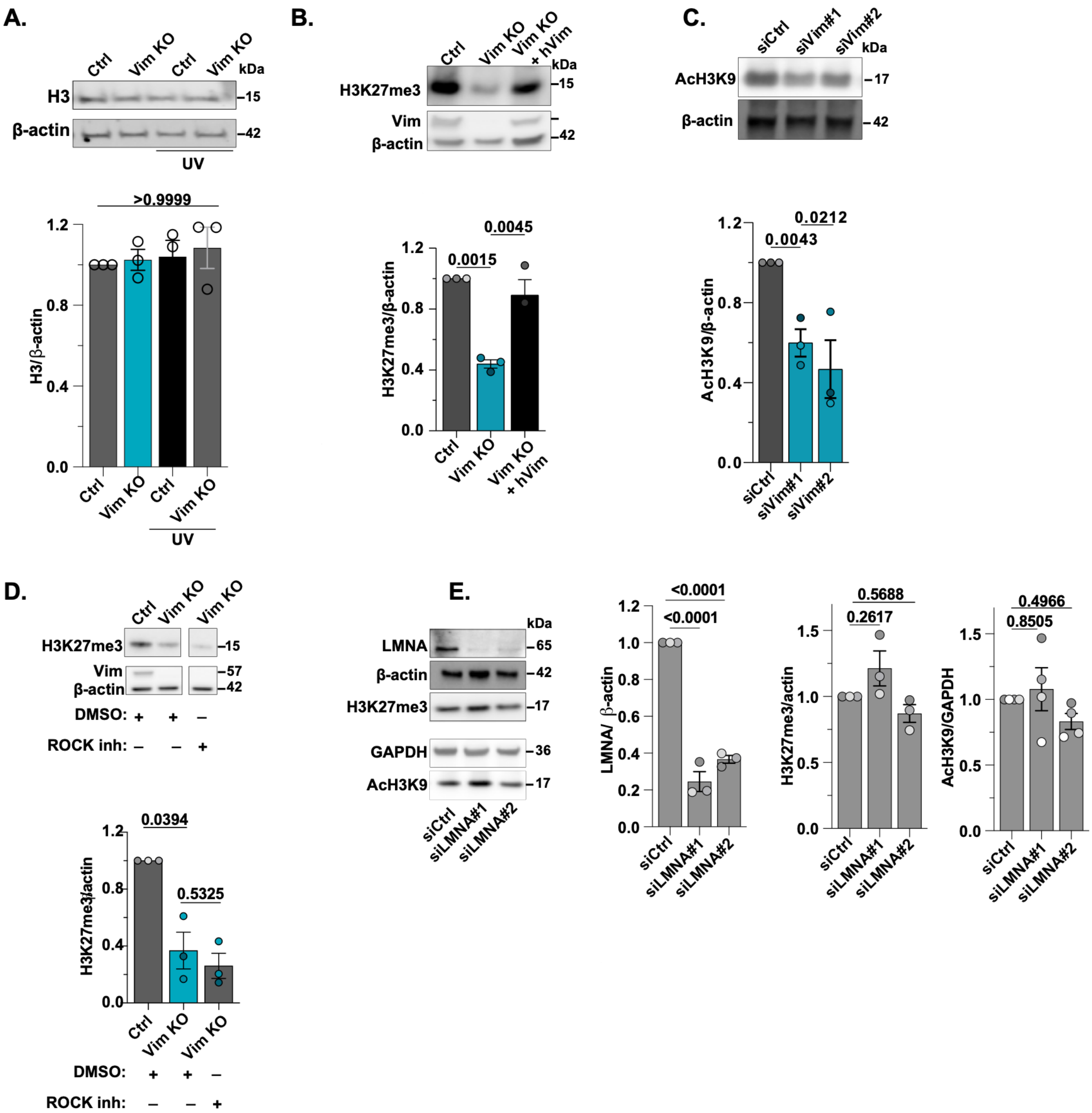

Appendix Figure S4.

A. U251-MG cells depleted or not for vimentin were irradiated with 210 mJ/cm² UV light, lysed after 24h, H3 protein levels were analysed by western blot and quantified using Fiji. Data were acquired from three independent experiments and analysed by one-way ANOVA. Error bars represent mean ± SEM.

B. H3K27me3 expression in control, Vim KO and Vimentin depleted cells overexpressing human vimentin were analysed by western blot, and relative expression was quantified using Fiji. Data were acquired from three independent experiments and analysed by one-way ANOVA. Error bars represent mean ± SEM.

C. U251-MG cells were transfected with siRNAs targeting vimentin. Levels of acH3K9 were analysed by western blot, and relative expression was quantified using Fiji. Data were acquired from three independent experiments and analysed by two-tailed *t*-test. Error bars represent mean ± SEM.

D. U251-MG cells depleted or not for vimentin were treated with either DMSO (vehicle) or ROCK inhibitor (5 μM Y27632.) for 4h). Levels of H3K27me3 and analysed by western blot and relative expression was quantified using Fiji. Data were acquired from three independent experiments and analysed by two-tailed *t*-test. Error bars represent mean ± SEM.

E. U251-MG cells were transfected with siRNAs targeting vimentin. Levels of lamin A/C, H3K27me3 and acH3K9 were analysed by western blot, and relative expression was quantified using Fiji. Data were acquired from three independent experiments and analysed by one-way ANOVA. Error bars represent mean ± SEM.

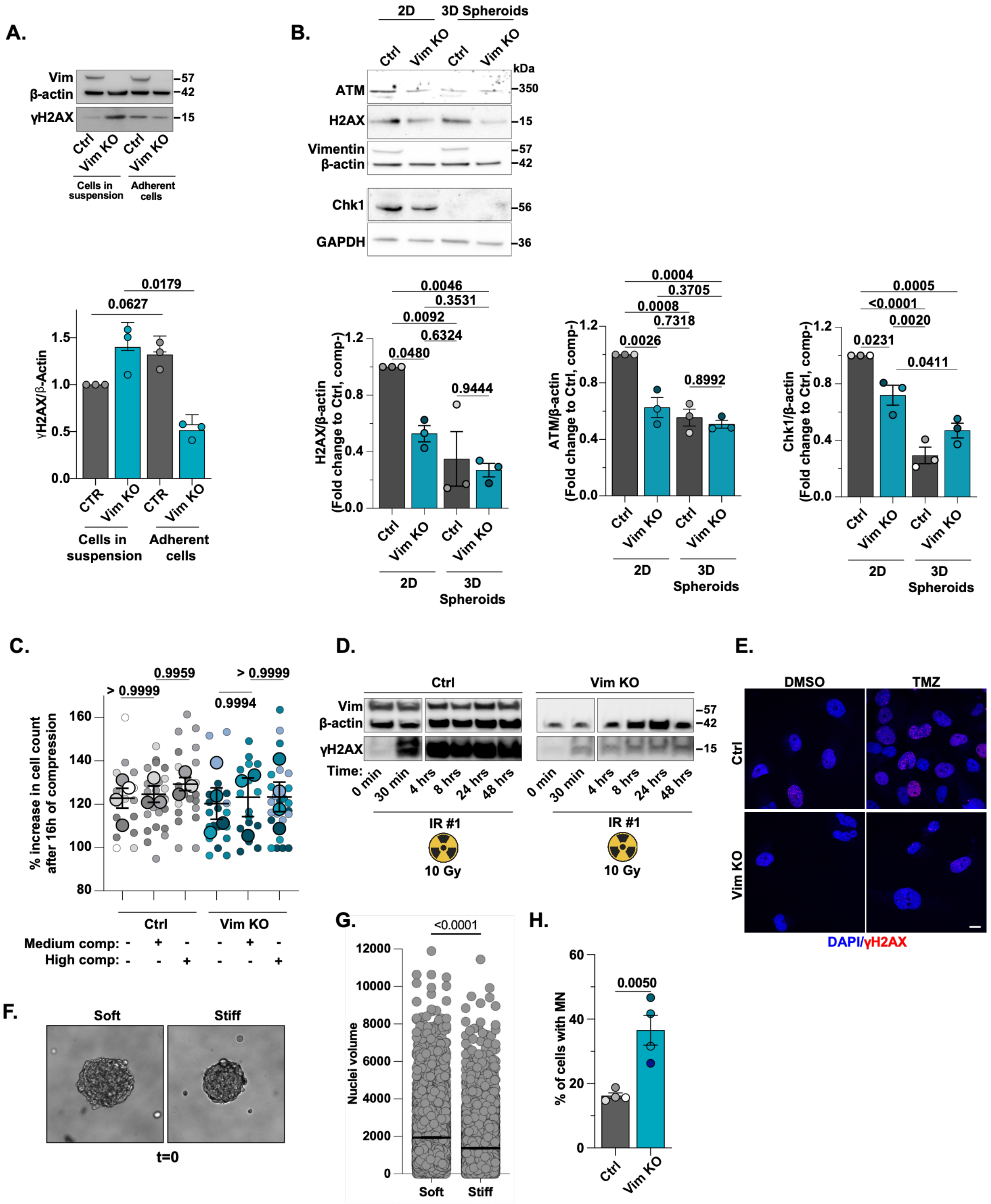

Appendix Figure S5.

A. Expression of γH2AX in control and Vim KO U251-MG cells cultured as suspension cells or in 2D was analysed by western blot and relative expression was quantified using Fiji. Data were acquired from three independent experiments and analysed using a two-tailed *t*-test. Error bars represent mean ± SEM.

B. Expression of ATM, Chk1 and H2AX in control and Vim KO U251-MG cells cultured as sparse 2D cells or as spheroids was analysed by western blot and relative expression was

quantified using Fiji. Data were acquired from three independent experiments and analysed using a two-tailed *t*-test. Error bars represent mean ± SEM.

C. Cell proliferation was quantified after 16 h under no, medium, or high compression. SuperPlots show the percentage increase in cell number. Individual data points represent fields of view of Ctrl (grey) or Vim KO (blue). Larger dots indicate the mean of each independent experiment. Data were acquired from three or four independent experiments and analysed using a one-way ANOVA test. Error bars represent mean ± SEM.

D. U251-MG cells depleted or not for vimentin were irradiated with a single dose of 10Gy X-rays and γH2AX expression was analysed by western blot at the indicated time points.

E. Representative images of U251-MG Ctrl or vim KO cells treated with either DMSO (vehicle) or temozolomide for 48h and stained for γH2AX and DAPI to visualise the nuclei (scale bar 10 µm).

F. Representative bright-field images of gliospheres embedded in soft or stiff gel at t=0.

G. Nuclear volume of gliospheres after 3 days embedded in soft or stiff gels, quantified with IMARIS software and analysed by two-tailed *t*-test. Error bars represent mean ± SEM.

H. Percentage of cells with micronuclei were scored in U251-MG cells ctrl or vim KO cells. Data were acquired from four independent experiments and analysed using two-tailed *t*-test. Error bars represent mean ± SEM.
