# Supplementary material for: Vimentin loss inhibits DNA damage responses and promotes cancer cell survival
Source: EMBO Rep. 2026 Sep 4;27(19):5561–81. doi: 10.1038/s44319-026-00913-2 (PMC13649785; doi:10.1038/s44319-026-00913-2)
Supplement: Supplementary file 2 [file 44319_2026_913_MOESM2_ESM.pdf]

Vimentin Loss Inhibits DNA Damage Responses and Promotes Cancer Cell Survival.

Elvira Infante, Emmanuel Terriac, Matthieu Gélin, Vanessa Roca, Saline Jabre, Hugo Siegfried, David Pereira, Hugo Varet, Sara Khalilian, Atef Asnacios, Emma van Bodegraven, and Sandrine Etienne-Manneville

Corresponding author(s): Elvira Infante (elvira.infante@kcl.ac.uk) , Sandrine Etienne-Manneville (sandrine.etienne-manneville@pasteur.fr)

Review Timeline:

Transfer Date:	14th Mar 26
Editorial Decision:	17th Mar 26
Revision Received:	18th May 26
Editorial Decision:	10th Jul 26
Revision Received:	22nd Jul 26
Accepted:	13th Aug 26

Editor: Kurt Weir

Transaction Report: This manuscript was transferred to EMBO Reports following arbitrating peer review at The EMBO Journal, which was preceded by full peer review and revision at another journal.

(Note: With the exception of the correction of typographical or spelling errors that could be a source of ambiguity, letters and reports are not edited. Depending on transfer agreements, referee reports obtained elsewhere may or may not be included in this compilation. Referee reports are anonymous unless the Referee chooses to sign their reports.)

The manuscript entitled "Vimentin loss inhibits DNA damage responses (DDR) and promotes cancer cell survival" explores a potential connection between vimentin and the DNA damage response machinery. In this manuscript the authors make a series of observations regarding the expression and function of genes related to DDR, chromatin modifications, and cell survival in the absence of Vimentin. These observations on their Vimentin knock-out cell lines are certainly very intriguing and hold a great potential. However, even though it can be acknowledged that a lot of nice work has been done, the manuscript remains mostly a series of observations without any mechanistic link to each other. I believe the authors still have not addressed the main point of concern: How does "mechanochemical" vimentin regulate the DDR system? The authors mention that this effect could be explained by the loss of both heterochromatin and euchromatin markers. However, this is a very loose connection at best. If the authors would like to claim this, they should have performed ATAC seq and compared the accessibility of the highlighted genes in control compared to Vimentin KO and upon re-introducing vimentin in the knock out setting.

Strikingly, the authors performed a rescue experiment in which they only tested the DNA damage accumulation in the absence of vimentin. I believe it would have been essential to perform rescue experiments on their other observations too, namely involvement of the other filamentous structures like microtubules on the nuclear compression, chromatin modifications, and survivability.

There were also experimental approaches that seem not ideal. The authors claim the microtubule network is the main driver for nuclear compression when the vimentin shielding is missing, while this is not the case for actomyosin contractility. However, while they show that there is the same amount of F-actin associated with the nucleus, that does not mean that they apply the same amount of force on it. It is surprising that they did not inhibit actin polymerization and measure the nuclear height like they did with the nocodazole treatment. Also, I am not entirely sure if ROCK inhibition is the best way to interrogate actomyosin contractility. Maybe blebbistatin in order to inhibit the ATPase activity of the myosins? ROCK inhibition could also have effects on actin polymerization dynamics through LIMK and cofilin.

The authors claim that loss of vimentin leads to the bypassing of certain cell cycle checkpoints and evasion of apoptosis, which they do not really explore. The authors should have taken advantage of reporter systems such as FUCCI and dyes such as the live cell imaging caspase dye BioTracker NucView (SCT100) in the presence or absence of Vimentin.

To summarize, this manuscript holds a high potential, but at its current state it is a series of observations which the authors try to connect but they lack the proof to do so.

1st Editorial Decision 17th Mar 2026

Dear Dr. Etienne-Manneville

Thank you for the submission of your research manuscript to our journal.

As you have seen, the arbitrator acknowledges that the findings are interesting and that the conclusions are overall supported by the data presented but they also raise a number of concerns and have suggestions how to further strengthen the data.

For consideration at EMBO Reports, we will require:
- Test the potential role of actin in nuclear compression in line with the arbitrator's comments.
- Incorporate additional evidence to investigate actomyosin contractility in line with the arbitrator's comments.

For consideration at EMBO Reports, we do not require additional clarification of the mechanisms linking nuclear compression and DDR pathway expression or checkpoint evasion.

Given these constructive comments, we would like to invite you to revise your manuscript with the understanding that the arbitrator concerns detailed above must be fully addressed and their suggestions taken on board. Please address all referee concerns in a complete point-by-point response. Acceptance of the manuscript will depend on a positive outcome of a second round of review. It is EMBO Reports policy to allow a single round of revision only and acceptance or rejection of the manuscript will therefore depend on the completeness of your responses included in the next, final version of the manuscript.

We realize that it is difficult to revise to a specific deadline. In the interest of protecting the conceptual advance provided by the work, we recommend a revision within 3 months (June 17th). Please discuss the revision progress ahead of this time with the editor if you require more time to complete the revisions.

I am also happy to discuss the revision further via e-mail or a video call, if you wish.

You can either publish the study as a short report or as a full article. For short reports, the revised manuscript should not exceed 27,000 characters (including spaces but excluding materials & methods and references) and 5 main plus 5 expanded view figures. The results and discussion sections must further be combined, which will help to shorten the manuscript text by eliminating some redundancy that is inevitable when discussing the same experiments twice. For a normal article there are no length limitations, but it should have more than 5 main figures and the results and discussion sections must be separate. In both cases, the entire materials and methods must be included in the main manuscript file.

================
IMPORTANT NOTE:

We perform an initial quality control of all revised manuscripts before re-review. Your manuscript will FAIL this control and the handling will be delayed IN CASE the following APPLIES:

1) A data availability section providing access to data deposited in public databases is missing. If you have not deposited any data, please add a sentence to the data availability section that explains that.

2) Your manuscript contains statistics and error bars based on n=2. Please use scatter blots in these cases. No statistics should be calculated if n=2.

When submitting your revised manuscript, please carefully review the instructions that follow below. Failure to include requested items will delay the evaluation of your revision.

================

When submitting your revised manuscript, we will require:

1) a .docx formatted version of the manuscript text (including legends for main figures, EV figures and tables). Please make sure that the changes are highlighted to be clearly visible.

2) individual production quality figure files as .eps, .tif, .jpg (one file per figure).
Please download our Figure Preparation Guidelines (figure preparation pdf) from our Author Guidelines pages https://www.embopress.org/page/journal/14693178/authorguide for more info on how to prepare your figures.

3) a .docx formatted letter INCLUDING the reviewers' reports and your detailed point-by-point responses to their comments. As part of the EMBO Press transparent editorial process, the point-by-point response is part of the Review Process File (RPF),

which will be published alongside your paper.

4) a complete author checklist, which you can download from our author guidelines (<https://www.embopress.org/page/journal/14693178/authorguide>). Please insert information in the checklist that is also reflected in the manuscript. The completed author checklist will also be part of the RPF.

5) Please note that all corresponding authors are required to supply an ORCID ID for their name upon submission of a revised manuscript (<https://orcid.org/>). Please find instructions on how to link your ORCID ID to your account in our manuscript tracking system in our Author guidelines (<https://www.embopress.org/page/journal/14693178/authorguide#authorshipguidelines>)

6) We replaced Supplementary Information with Expanded View (EV) Figures and Tables that are collapsible/expandable online. A maximum of 5 EV Figures can be typeset. EV Figures should be cited as 'Figure EV1, Figure EV2" etc... in the text and their respective legends should be included in the main text after the legends of regular figures.

- For the figures that you do NOT wish to display as Expanded View figures, they should be bundled together with their legends in a single PDF file called *Appendix*, which should start with a short Table of Content. Appendix figures should be referred to in the main text as: "Appendix Figure S1, Appendix Figure S2" etc. See detailed instructions regarding expanded view here: <https://www.embopress.org/page/journal/14693178/authorguide#expandedview>

- Additional Tables/Datasets should be labeled and referred to as Table EV1, Dataset EV1, etc. Legends have to be provided in a separate tab in case of .xls files. Alternatively, the legend can be supplied as a separate text file (README) and zipped together with the Table/Dataset file.

7) Before submitting your revision, primary datasets (and computer code, where appropriate) produced in this study need to be deposited in an appropriate public database (see < https://www.embopress.org/page/journal/14693178/authorguide#dataavailability>).

Please remember to provide a reviewer password if the datasets are not yet public.

The accession numbers and database should be listed in a formal "Data Availability " section (placed after Materials & Method) that follows the model below (see also < https://www.embopress.org/page/journal/14693178/authorguide#dataavailability>). Please note that the Data Availability Section is restricted to new primary data that are part of this study.

Data availability

The datasets (and computer code) produced in this study are available in the following databases:

- RNA-Seq data: Gene Expression Omnibus GSE46843 (https://www.ncbi.nlm.nih.gov/geo/query/acc.cgi?acc=GSE46843)
- [data type]: [name of the resource] [accession number/identifier/doi] ([URL or identifiers.org/DATABASE:ACCESSION])

*** Note - All links should resolve to a page where the data can be accessed. ***

8) At EMBO Press we ask authors to provide source data for the main manuscript figures. You will receive a separate email with instructions for providing source data with your revised manuscript, including how to upload and organize the files.

Additional information on source data and instruction on how to label the files are available <https://www.embopress.org/page/journal/14693178/authorguide#sourcedata>

9) The journal requires a statement specifying whether or not authors have competing interests (defined as all potential or actual interests that could be perceived to influence the presentation or interpretation of an article). In case of competing interests, this must be specified in your disclosure statement. Further information: https://www.embopress.org/competing-interests

10) Figure legends and data quantification:
The following points must be specified in each figure legend:

- the name of the statistical test used to generate error bars and P values,
- the EXACT p-values,
- the number (n) of independent experiments (please specify technical or biological replicates) underlying each data point,
- the nature of the bars and error bars (s.d., s.e.m.)

- If the data are obtained from n {less than or equal to} 5, show the individual data points in addition to the SD or SEM.
- If the data are obtained from n {less than or equal to} 2, use scatter blots showing the individual data points.

Discussion of statistical methodology can be reported in the materials and methods section, but figure legends should contain a basic description of n, P and the test applied.

See also the guidelines for figure legend preparation:
https://www.embopress.org/page/journal/14693178/authorguide#figureformat

- Please also include scale bars in all microscopy images and define their size in the legend, not the image.

11) Our journal encourages inclusion of *data citations in the reference list* to directly cite datasets that were re-used and obtained from public databases. Data citations in the article text are distinct from normal bibliographical citations and should directly link to the database records from which the data can be accessed. In the main text, data citations are formatted as follows: "Data ref: Smith et al, 2001" or "Data ref: NCBI Sequence Read Archive PRJNA342805, 2017". In the Reference list, data citations must be labeled with "[DATASET]". A data reference must provide the database name, accession number/identifiers and a resolvable link to the landing page from which the data can be accessed at the end of the reference. Further instructions are available at <https://www.embopress.org/page/journal/14693178/authorguide#referencesformat>.

12) All Materials and Methods need to be described in the main text using our 'Structured Methods' format. According to this format, the Methods section includes a Reagents and Tools Table (listing key reagents, experimental models, software and relevant equipment and including their sources and relevant identifiers) followed by a Methods and Protocols section describing the methods, ideally using a step-by-step protocol format. The aim is to facilitate adoption of the methodologies across labs. Please download and fill our Reagents and Tools Table template (.docx), which you can find in our author guidelines: https://www.embopress.org/page/journal/14693178/authorguide#structuredmethods.
When submitting your revised manuscript, please do not include the Reagents and Tools Table in the Methods section of the manuscript but upload it as a separate file choosing the file type "Reagent Table".
An example of a Method paper with Structured Methods can be found here:
https://www.embopress.org/doi/10.15252/msb.20178071.

13) As part of the EMBO publication's Transparent Editorial Process, EMBO Reports publishes online a Review Process File to accompany accepted manuscripts. This File will be published in conjunction with your paper and will include the referee reports, your point-by-point response and all pertinent correspondence relating to the manuscript.

You are able to opt out of this by letting the editorial office know (emboreports@embo.org). If you do opt out, the Review Process File link will point to the following statement: "No Review Process File is available with this article, as the authors have chosen not to make the review process public in this case."

We would also welcome the submission of cover suggestions, or motifs to be used by our Graphics Illustrator in designing a cover.

I look forward to seeing a revised form of your manuscript when it is ready and please let me know if you have questions or comments regarding the revision.

You can use this link to submit your revision: https://embor.msubmit.net/cgi-bin/main.plex

Yours sincerely,

Kurt Weir
Editor
EMBO Reports

We thank the arbitrator for the constructive comments and the editors for their guidance. As recommended by the editor, we focused the revision on additional experiments clarifying the role of actin in controlling nuclear shape in vimentin-depleted cells.

1- **Test the potential role of actin in nuclear compression in line with the arbitrator's comments**.

R: Low-dose cytochalasin D (100 or 200 nM) to perturb actin polymerization did not restore Vim KO nuclear height or aspect ratio (new Figures S3F–G), indicating that actin dynamics are not a primary determinant of nuclear morphology in this context. Higher doses (1–2 μM) caused cell detachment, precluding reliable shape analysis. To isolate the contribution of adhesion, we analyzed cells in suspension, which altered γH2AX levels in Vim KO cells (new Figure S5A). Together, these data support the conclusion that adhesion-dependent mechanical constraints, rather than actin filament organization per se, govern nuclear compression and associated DDR signaling in vimentin-deficient cells (see our discussion p12).

2-Incorporate additional evidence to investigate actomyosin contractility in line with the arbitrator's comments

R: we complemented the ROCK inhibition experiments with blebbistatin treatment to inhibit myosin II activity. As with ROCK inhibitors, blebbistatin did not rescue the 'compressed' nuclear morphology of vimentin KO cells (new Figures S3G-E), indicating that actomyosin-generated contractile forces are not the primary drivers of nuclear deformation in this setting.

Dear Dr. Infante

Thank you for the submission of your revised manuscript to EMBO reports. We have now received the arbitrator report that is copied below my signature.

As you can see, the arbitrator finds that the study is significantly improved during revision and recommends publication. Before I can accept the manuscript, I need you to address some minor points below:

- As a standard procedure, we edit the abstract of manuscripts to make them more accessible to a general readership. Please find the suggested version below my signature, which has been edited to meet our word limit.

- Please put the manuscript sections in the following order: Title page - Abstract - Introduction - Results - Discussion - Methods - Data Availability - Acknowledgments - Disclosure Statement & Competing Interests - References - Figure Legends - (Main Tables with legends if applicable) - Expanded View Figure Legends.

- Please rename the Materials and Methods section to Methods.

- Please rename the Competing interests section to Disclosure and Competing Interests Statement and remove the Ethics secion.

- Please remove the sentence "All the raw data and code generated during this study are available from the corresponding author on reasonable request" from the data availability section.

- Funding should be part of Acknowledgements, no separate subsection heading is needed.

- CRediT has replaced the traditional author contributions section because it offers a systematic machine readable author contributions format that allows for more effective research assessment. Please remove the Author Contributions section from the manuscript and use the free text boxes beneath each contributing author's name in our online systems to add specific details on the author's contribution. More information is available in our guide to authors.

- Please update the author names in the manuscript to match the format in the system, namely First Name Last Name.

- Please adjust the format of the reference list and of the in-text citations according to EMBO Journal format (alphabetical order, DOIs only used for preprints and datasets that have not been published yet). Please refer to our Guide to Authors for additional information on EMBO J reference format.

- Please also adjust the format for citation of preprints and for public datasets as specified in our author guidelines:
Preprints:
The citation in the text should be: "(preprint: NAME1 et al, YEAR)"
The citation in the reference list: "Author NAME1, Author NAME2, ... (YEAR) article title. PLATFORM NAME, doi: XXX [PREPRINT]"
Datasets:
Hörnberg E, Ylitalo EB, Crnalic S, Antti H, Stattin P, Widmark A, Bergh A, Wikström P (2011) Gene Expression Omnibus GSE29650 (https://www.ncbi.nlm.nih.gov/geo/query/acc.cgi?acc=GSE29650). [DATASET]
Hörnberg E, Ylitalo EB, Crnalic S, Antti H, Stattin P, Widmark A, Bergh A, Wikström P (2011) Expression of androgen receptor splice variants in prostate cancer bone metastases is associated with castration-resistance and short survival. PLoS One 6: e19059
In the main text, these datasets should be cited with the prefix "Data ref:" to distinguish them from the reference to the original article that reported the dataset. Example:
"...were grouped based on the relative levels of AR-Vs expressed, mainly AR-V7 (Hörnberg et al, 2011; Data ref: Hörnberg et al, 2011)."
Please, refer to our detailed online guidelines for further information:
https://www.embopress.org/page/journal/14602075/authorguide#referencesformat

- Please include a Reagents and Tools Table (listing key reagents, experimental models, software, and relevant equipment and including their sources and relevant identifiers), uploaded as separate file. More information on how to adhere to this format as well as downloadable templates (.doc) for the Reagents and Tools Table can be found in our author guidelines (section 'Structured Methods'): https://www.embopress.org/page/journal/14602075/authorguide#structuredmethods

- Thank you for uploading source data to BioStudies. To facilitate source data checks, please make sure to provide all the necessary Source data files to our system and list them in the Source Data checklist file. Completed SD checklist should be uploaded as Related Manuscript File. Source data files need to be saved in a scheme one figure/folder and then uploaded as

.zip files. E.g. all the Source data files for figure 1 need to be saved in a single folder and this needs to be zipped and then uploaded as "SD figure 1.zip" file. For EV and/or appendix figures, ZIP together all source data.

- Please fill in the source data category in the Source Data Checklist.

- Please include a response for cell D59 in the Author Checklist.

- Please include the funder NWO VI.Veni.212.117 in the system.

- Please correct callouts of the Appendix figures to include "Appendix".

- Please do not use "Supplementary" in movie callouts.

- Thank you for providing a Supplementary Figures file. Please update the nomenclature throughout the file (Appendix for [Ms Title], Appendix Figures instead of Supplementary Figures, Appendix Figure S1, etc.). The author information is not needed on the title page. Please include a table of contents that lists each item with its page number. Please remove the movie legends from the Appendix file. Please remove the list of Antibodies to the Reagents table.

- Please update the nomenclature for movies in all places to Movie EV1, etc. The legend for each movie should be provided in a readme.txt file and zipped together with its corresponding movie so that we have one folder per movie upload.

- Please note that EMBO press papers are accompanied online by:
A) a short (2 sentences) summary of the findings and their significance,
B) 2-5 short bullet points highlighting the key results, and
C) a synopsis image in .jpg or .png format that is exactly 550 pixels wide and 300 pixels high. Please note that the text needs to be legible at the final size. Please upload this information along with your revised manuscript (the text for A and B should be provided in one separate Word file uploaded as Cover Art/Synopsis Image).

- It appears that there is repeated use of an actin blot in Appendix Figure 2A. Can you explain why this is so? If the targets to which it is being compared came from the same blot, please indicate so in the figure legend.

- Please include a statement on blinding in the Methods section even if no blinding was performed.

- Our production/data editors have asked you to clarify several points in the figure legends (see below). Please incorporate these changes in the manuscript and return the revised file with tracked changes with your final manuscript submission.
- Please note that the exact p values are not provided in the legends of 2G; 3B; 4J; 6A; 7M,O; Supplementary figure 2C,G; Supplementary figure 4E; Supplementary figure 5B,G.
- Please indicate the statistical test used for data analysis in the legends of figures 1C; Supplementary figure 5G;
- Please note that information related to n is missing in the legends 1C.
- Please note that the error bars are not defined in the legends of figures 2A,B,D,E,F,G,I; 3A-E; 4C,E,G,I,J,K,L,N-Q; 5A-F; 6A-C; 7B,D,E,J,K,M,O; Supplementary figure 2A,C,D,F,G,H,I; Supplementary figure 3A,B,D,E,F,G; Supplementary figure 4A-E; Supplementary figure 5A,B,C,H.
- 1. Please note that scale bar and its definition are missing for Supplementary figure 3C.
- Please doublecheck that legends in the Appendix file meet the requirements for the main text figures.

Kurt Weir
Editor
EMBO Reports

Vimentin intermediate filaments are a hallmark of aggressive tumors and are widely linked to invasion and EMT, yet how vimentin-dependent mechanics shape genome maintenance and therapy response is unclear. Here we show that vimentin, particularly under compressive load, promotes DNA repair competence. In contrast, vimentin negative cells show impaired DNA damage sensing and downstream signaling, ultimately leading to decreased apoptosis and promoting cell survival under genotoxic stress at the expense of genomic stability. Using controlled cell compression together with genetic and pharmacological perturbations, we find that loss of vimentin of glioblastoma cells limit the expression and activity of core repair pathways because of induced nuclear mechanical compression. Relieving nuclear compression restores DNA damage accumulation and repair kinetics. Functionally, suppression of DNA damage responses enhances survival after clinically relevant DNA-damaging treatments, including temozolomide, X-Ray radiation and cell invasion through tight spaces. These findings invert the prevailing view that vimentin's contribution to tumor progression stems from enhanced migration and identify a mechanochemical vimentin-nucleus axis that tunes DNA damage responses to favor therapy tolerance and genome evolution.

Referee #1:

The manuscript "Vimentin loss inhibits DNA damage responses and promotes cancer cell survival" by Infante et al. investigates

the role of heterogeneous expression of the mechano-protective cytoskeletal protein vimentin in glioblastoma (GBM), an aggressive and currently incurable brain cancer.

Combining cell biology, biomechanical approaches, and transcriptional analyses, the authors show that vimentin protects cells from mechanical compression and that its loss impairs DNA damage repair enhancing cell proliferation and survival when exposed to genotoxic stress. These data provide new insight into mechanisms that may contribute to cancer progression, treatment responses, and resistance to DNA-damaging therapies.

This is a well-executed cell biology study that presents a coherent mechanistic framework with clear relevance to GBM biology. The authors have addressed the concerns raised by the four reviewers during the initial submission to The EMBO Journal by providing appropriate responses and additional experimental evidence included in the revised manuscript.

Following the transfer of the manuscript to EMBO reports, the authors were asked to address two specific issues. Both have now been satisfactorily resolved through additional experiments that demonstrate that actin does not play a role in nuclear shape/compression in the absence of vimentin.

All editorial and formatting issues were resolved by the authors.

Dr. Elvira Infante
King's College London
Randall Centre for Cell and Molecular Biophysics
New Hunt's House
Guy's Campus
London SE1 1UL
United Kingdom

Dear Dr. Infante,

I am pleased to inform you that your manuscript has been accepted for publication in EMBO reports. Your manuscript will be processed for publication by EMBO Press. It will be copy edited and you will receive page proofs prior to publication. Please note that you will be contacted by Springer Nature Author Services to complete licensing and payment information.

You may qualify for financial assistance for your publication charges - either via a Springer Nature fully open access agreement or an EMBO initiative. Check your eligibility: https://link.springer.com/journal/44319/how-to-publish-with-us

Should you be planning a Press Release on your article, please notify the production team via this form in order to coordinate publication and release dates: https://forms.cloud.microsoft/e/eH4A8JVP6g

If you have any questions, please do not hesitate to contact the Editorial Office. Thank you for your contribution to EMBO Reports.

Yours sincerely,

Kurt Weir
Editor
EMBO Reports

>>> Please note that it is EMBO Reports policy for the transcript of the editorial process (containing referee reports and your response letter) to be published as an online supplement to each paper. If you do NOT want this, you will need to inform the Editorial Office via email immediately. More information is available here: https://link.springer.com/partners/embo-press/editorial-policies#Peer%20review

EMBO Press Author Checklist

Corresponding Author Name: Elvira Infante and Sandrine Etienne-Manneville
Journal Submitted to: EMBO Reports
Manuscript Number: EMBOR-2026-64038-T

USEFUL LINKS FOR COMPLETING THIS FORM
The EMBO Journal - Author Guidelines
EMBO Reports - Author Guidelines
Molecular Systems Biology - Author Guidelines
EMBO Molecular Medicine - Author Guidelines

Reporting Checklist for Life Science Articles (updated January
This checklist is adapted from Materials Design Analysis Reporting (MDAR) Checklist for Authors. MDAR establishes a minimum set of requirements in transparent reporting in the life sciences (see Statement of Task: 10.31222/osf.io/9sm4x). Please follow the journal's guidelines in preparing your
Please note that a copy of this checklist will be published alongside your article.

Abridged guidelines for figures
1. Data
The data shown in figures should satisfy the following conditions:
- the data were obtained and processed according to the field's best practice and are presented to reflect the results of the experiments in an accurate and unbiased manner.
- ideally, figure panels should include only measurements that are directly comparable to each other and obtained with the same assay.
- plots include clearly labeled error bars for independent experiments and sample sizes. Unless justified, error bars should not be shown for technical
- if n<5, the individual data points from each experiment should be plotted. Any statistical test employed should be justified.
- Source Data should be included to report the data underlying figures according to the guidelines set out in the authorship guidelines on Data

2. Captions
Each figure caption should contain the following information, for each panel where they are relevant:
- a specification of the experimental system investigated (eg cell line, species name).
- the assay(s) and method(s) used to carry out the reported observations and measurements.
- an explicit mention of the biological and chemical entity(ies) that are being measured.
- an explicit mention of the biological and chemical entity(ies) that are altered/varied/perturbed in a controlled manner.
- the exact sample size (n) for each experimental group/condition, given as a number, not a range;
- a description of the sample collection allowing the reader to understand whether the samples represent technical or biological replicates (including how many animals, litters, cultures, etc.).
- a statement of how many times the experiment shown was independently replicated in the laboratory.
- definitions of statistical methods and measures:
  - common tests, such as t-test (please specify whether paired vs. unpaired), simple χ2 tests, Wilcoxon and Mann-Whitney tests, can be unambiguously identified by name only, but more complex techniques should be described in the methods section;
  - are tests one-sided or two-sided?
  - are there adjustments for multiple comparisons?
  - exact statistical test results, e.g., P values = x but not P values < x;
  - definition of 'center values' as median or average;
  - definition of error bars as s.d. or s.e.m.

Please complete ALL of the questions below.
Select "Not Applicable" only when the requested information is not relevant for your study.

Materials

Newly Created Materials	Information included in the manuscript?	In which section is the information available? (Reagents and Tools Table, Materials and Methods, Figures, Data Availability Section)
New materials and reagents need to be available; do any restrictions apply?	Not Applicable	

Antibodies	Information included in the manuscript?	In which section is the information available? (Reagents and Tools Table, Materials and Methods, Figures, Data Availability Section)
For **antibodies** provide the following information:<br>- Commercial antibodies: RRID (if possible) or supplier name, catalogue number and or/clone number<br>- Non-commercial: RRID or citation	Yes	Manuscript expanded view content

DNA and RNA sequences	Information included in the manuscript?	In which section is the information available? (Reagents and Tools Table, Materials and Methods, Figures, Data Availability Section)
**Short novel DNA or RNA including primers, probes**: provide the sequences.	Not Applicable	

Cell materials	Information included in the manuscript?	In which section is the information available? (Reagents and Tools Table, Materials and Methods, Figures, Data Availability Section)
**Cell lines:** Provide species information, strain. Provide accession number in repository **OR** supplier name, catalog number, clone number, and/**OR** RRID.	Yes	Materials and Methods
**Primary cultures:** Provide species, strain, sex of origin, genetic modification status.	Yes	Materials and Methods
Report if the cell lines were recently **authenticated** (e.g., by STR profiling) and tested for mycoplasma contamination.	Yes	Materials and Methods

Experimental animals	Information included in the manuscript?	In which section is the information available? (Reagents and Tools Table, Materials and Methods, Figures, Data Availability Section)
**Laboratory animals or Model organisms:** Provide species, strain, sex, age, genetic modification status. Provide accession number in repository **OR** supplier name, catalog number, clone number, **OR** RRID.	Not Applicable	
**Animal observed in or captured from the field:** Provide species, sex, and age where possible.	Not Applicable	
Please detail **housing and husbandry conditions**.	Not Applicable	

Plants and microbes	Information included in the manuscript?	In which section is the information available? (Reagents and Tools Table, Materials and Methods, Figures, Data Availability Section)
**Plants:** provide species and strain, ecotype and cultivar where relevant, unique accession number if available, and source (including location for collected wild specimens).	Not Applicable	
**Microbes:** provide species and strain, unique accession number if available, and source.	Not Applicable	

Human research participants	Information included in the manuscript?	In which section is the information available? (Reagents and Tools Table, Materials and Methods, Figures, Data Availability Section)
If collected and within the bounds of privacy constraints report on age, sex and gender or ethnicity for all study participants.	Not Applicable	

Core facilities	Information included in the manuscript?	In which section is the information available? (Reagents and Tools Table, Materials and Methods, Figures, Data Availability Section)
If your work benefited from core facilities, was their service mentioned in the acknowledgments section?	Yes	

Design

Study protocol	Information included in the manuscript?	In which section is the information available? (Reagents and Tools Table, Materials and Methods, Figures, Data Availability Section)
If study protocol has been **pre-registered, provide DOI in the manuscript**. For clinical trials, provide the trial registration number **OR** cite DOI.	Not Applicable	
Report the **clinical trial registration number** (at ClinicalTrials.gov or equivalent), where applicable.	Not Applicable	

Laboratory protocol	Information included in the manuscript?	In which section is the information available? (Reagents and Tools Table, Materials and Methods, Figures, Data Availability Section)
Provide DOI OR other citation details if **external detailed step-by-step protocols** are available.	Not Applicable	

Experimental study design and statistics	Information included in the manuscript?	In which section is the information available? (Reagents and Tools Table, Materials and Methods, Figures, Data Availability Section)
Include a statement about **sample size** estimate even if no statistical methods were used.	Yes	Figures
Were any steps taken to minimize the effects of subjective bias when allocating animals/samples to treatment (e.g. **randomization procedure**)? If yes, have they been described?	Not Applicable	
Include a statement about **blinding** even if no blinding was done.	Not Applicable	
Describe **inclusion/exclusion criteria** if samples or animals were excluded from the analysis. Were the criteria pre-established?<br><br>If sample or data points were omitted from analysis, report if this was due to attrition or intentional exclusion and provide justification.	Not Applicable	
For every figure, are **statistical tests** justified as appropriate? Do the data meet the assumptions of the tests (e.g., normal distribution)? Describe any methods used to assess it. Is there an estimate of variation within each group of data? Is the variance similar between the groups that are being statistically compared?	Yes	

Sample definition and in-laboratory replication	Information included in the manuscript?	In which section is the information available? (Reagents and Tools Table, Materials and Methods, Figures, Data Availability Section)
In the figure legends: state number of times the experiment was **replicated** in laboratory.	Yes	Figures
In the figure legends: define whether data describe **technical or biological replicates**.	Not Applicable	

Ethics

Ethics	Information included in the manuscript?	In which section is the information available? (Reagents and Tools Table, Materials and Methods, Figures, Data Availability Section)
Studies involving **human participants**: State details of **authority granting ethics approval** (IRB or equivalent committee(s), provide reference number for approval.	Not Applicable	
Studies involving **human participants**: Include a statement confirming that **informed consent** was obtained from all subjects and that the experiments conformed to the principles set out in the WMA Declaration of Helsinki and the Department of Health and Human Services Belmont Report.	Not Applicable	
Studies involving **human participants:** For publication of **patient photos**, include a statement confirming that consent to publish was obtained.	Not Applicable	
Studies involving experimental **animals**: State details of **authority granting ethics approva**l (IRB or equivalent committee(s), provide reference number for approval. Include a statement of compliance with ethical regulations.	Not Applicable	
Studies involving **specimen and field samples:** State if relevant **permits** obtained, provide details of authority approving study; if none were required, explain why.	Not Applicable	

Dual Use Research of Concern (DURC)	Information included in the manuscript?	In which section is the information available? (Reagents and Tools Table, Materials and Methods, Figures, Data Availability Section)
Could your study fall under dual use research restrictions? Please check biosecurity documents and list of **select agents and toxins** (CDC): https://www.selectagents.gov/sat/list.htm	Not Applicable	
If you used a select agent, is the security level of the lab appropriate and reported in the manuscript?	Not Applicable	
If a study is subject to dual use research of concern regulations, is the name of the **authority granting approval and reference number** for the regulatory approval provided in the manuscript?	Not Applicable	

Reporting

The MDAR framework recommends adoption of discipline-specific guidelines, established and endorsed through community initiatives. Journals have their own policy about requiring specific guidelines and recommendations to complement MDAR.

Adherence to community standards	Information included in the manuscript?	In which section is the information available? (Reagents and Tools Table, Materials and Methods, Figures, Data Availability Section)
State if relevant guidelines or checklists (e.g., **ICMJE, MIBBI, ARRIVE, PRISMA**) have been followed or provided.	Not Applicable	
For **tumor marker prognostic studies,** we recommend that you follow the **REMARK** reporting guidelines (see link list at top right). See author guidelines, under 'Reporting Guidelines'. Please confirm you have followed these guidelines.	Not Applicable	
For **phase II and III randomized controlled trials**, please refer to the **CONSORT** flow diagram (see link list at top right) and submit the CONSORT checklist (see link list at top right) with your submission. See author guidelines, under 'Reporting Guidelines'. Please confirm you have submitted this list.	Not Applicable	

Data Availability

Data availability	Information included in the manuscript?	In which section is the information available? (Reagents and Tools Table, Materials and Methods, Figures, Data Availability Section)
Have **primary datasets** been deposited according to the journal's guidelines (see 'Data Deposition' section) and the respective accession numbers provided in the Data Availability Section?	Yes	Data availability
Were **human clinical and genomic datasets** deposited in a public access-controlled repository in accordance to ethical obligations to the patients and to the applicable consent agreement?	Not Applicable	
Are **computational models** that are central and integral to a study available without restrictions in a machine-readable form? Were the relevant accession numbers or links provided?	Not Applicable	
If publicly available data were reused, provide the respective **data citations in the reference list**.	Yes	Materials and Methods
